# Speed breeding: protocols, application and achievements

**DOI:** 10.3389/fpls.2025.1680955

**Published:** 2025-09-17

**Authors:** Andrey Olegovich Blinkov, Pavel Yuryevich Kroupin, Anna Ruslanovna Dmitrieva, Alina Alexandrovna Kocheshkova, Gennady Ilyich Karlov, Mikhail Georgievich Divashuk

**Affiliations:** Department of Applied Genetic Technologies, All-Russia Research Institute of Agricultural Biotechnology, Moscow, Russia

**Keywords:** speed breeding, pure lines, accelerated flowering, breeding, homozygous lines, dormancy breaking

## Abstract

One of the limiting factors in breeding and genetic research is the time required to develop pure lines. This is due, on the one hand, to the prolonged vegetative period of a single generation and, on the other hand, to the specifics of inbreeding, which typically requires 4–6 consecutive generations of self-pollination in plant material. Researchers have always sought approaches that enable the rapid development of homozygous plant lines. Consequently, methods such as greenhouse cultivation during the autumn-winter period, single-seed descent, shuttle breeding, embryo culture, and doubled haploid technology have been introduced into practice. All these methods have both advantages and limitations. One of the latest approaches facilitating a significant reduction in the vegetative period of plants is speed breeding (SB). This method is based on the application of factors that shorten the time from sowing to flowering, as well as techniques that accelerate the generative phase of development and overcome postharvest dormancy. This review provides a comprehensive list and characterization of all factors that influence the efficiency of speed breeding to varying degrees. Among the factors discussed that reduce the sowing-to-flowering period are photoperiod, light sources, spectral composition and light intensity, temperature, carbon dioxide levels, vernalization, mineral nutrition, substrate volume, mechanical shoot removal, and the use of plant growth regulators. To shorten the generative phase, the review summarizes the application of embryo culture and forced desiccation of immature seeds, along with methods to overcome postharvest dormancy. Additionally, applications of genetic approaches and genetic engineering for shortening generation time in speed breeding are described. The review also consolidates detailed protocols for approximately thirty crops. The high efficiency of speed breeding in reducing both the vegetative period per generation and the time required to develop pure lines has led to its increasing adoption in various research fields. This review highlights the application of speed breeding for hybridization and pure line development, introgression of target alleles, and genomic selection. A list of phenotypic traits exhibiting high correlation between controlled-environment and field conditions is provided.

## 1 Introduction

One of the limiting factors in the rapid development of commercial plant varieties and F_1_ hybrids is the extended duration of the breeding process. This challenge arises not only from the prolonged vegetative period of a single generation but also from the complexity of developing pure lines, which requires hybridization followed by approximately 4–6 years of inbreeding. In self-pollinating crops, the resulting lines must undergo several years of evaluation to assess their suitability as future varieties. In cross-pollinating crops, where heterotic hybrid breeding has gained popularity, the developed lines serve only as initial material for subsequent crosses to test their combining ability. Selected promising varieties and F_1_ heterotic hybrids require multi-year evaluation across diverse ecological conditions by authorities responsible for testing and protecting plant breeding achievements (Yu and Chung, 2021). The final stage, seed production, can also take several years. Thus, without modern methods to shorten the breeding cycle, the development of varieties or F_1_ hybrids in annual crops such as wheat, sunflower, and others may extend up to 15 years before market release (Watson et al., 2018; Jamali et al., 2020). An even greater challenge—and a more time-consuming one—is the creation of varieties or F_1_ hybrids in biennial and perennial crops (Van Nocker and Gardiner, 2014; D’Angelo and Goldman, 2019; Zhuzhzhalova et al., 2020).

**Figure 1:**
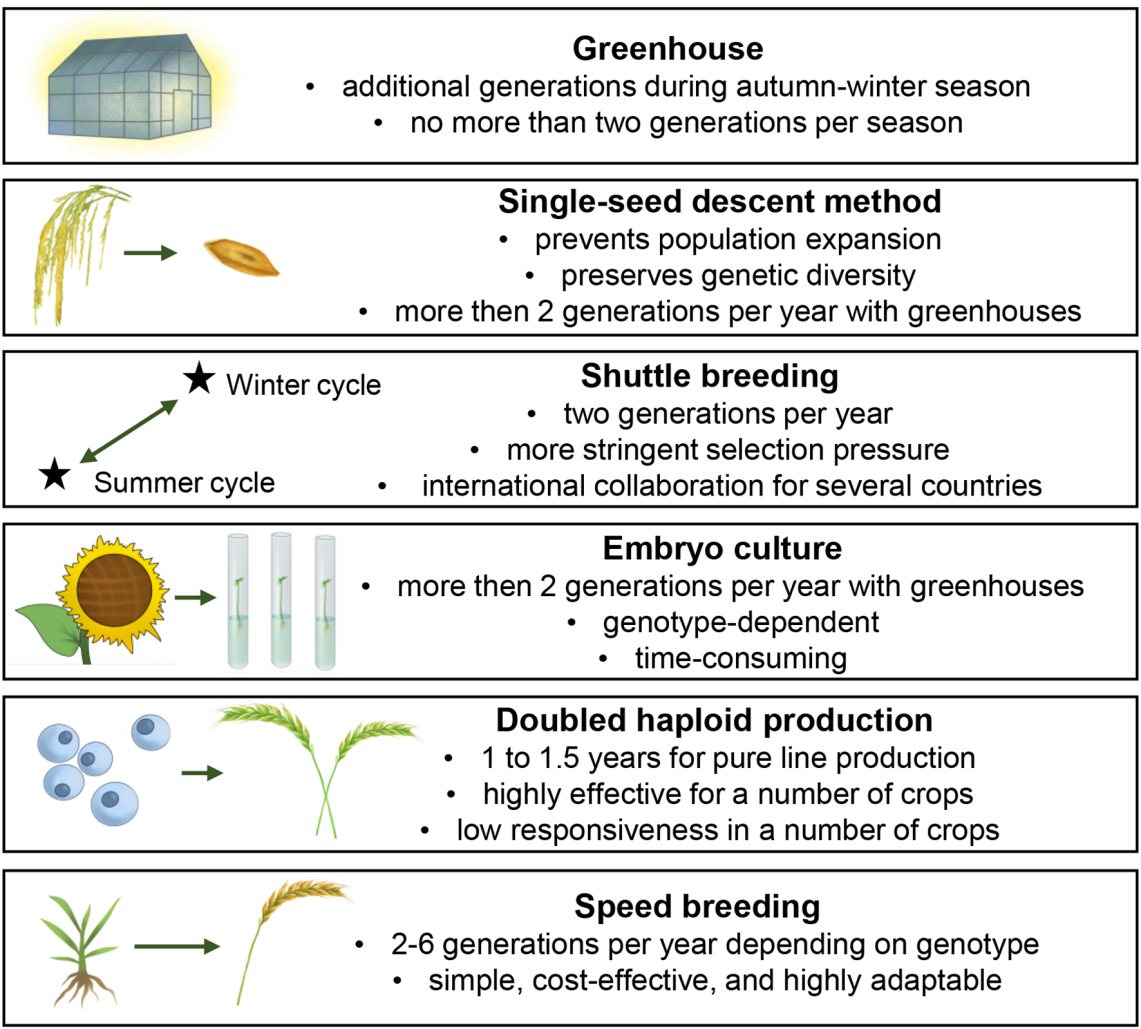
Different techniques aimed at the acceleration of pure line development.

Given the persistent need to develop plant varieties and hybrids resistant to changing biotic and abiotic stressors while improving yield and product quality, researchers have long sought methods to reduce the time required to obtain pure lines. Consequently, several techniques aimed at accelerating the development of homozygous forms have been integrated into breeding practices (**Figure 1**).

The first strategy to expedite breeding and research processes involved the use of greenhouses to cultivate additional generations during winter (McFadden and Brookings, 1917; Magruder, 1937). Although this approach yields no more than 1–2 generations per autumn-winter cycle, it remains widely employed today (Sandukhadze et al., 2016). Beyond greenhouses, off-season generations are also produced in controlled-environment facilities such as growth chambers and phytotrons (Volovik and Prologova, 2017).

In 1939, the single-seed descent (SSD) method was introduced to accelerate the development of homozygous lines. This technique involves advancing each generation by selecting only one seed per plant from a segregating population. While it prevents population expansion, it preserves genetic diversity. Importantly, SSD does not involve selective pressure during the inbreeding phase; instead, it focuses on rapidly generating pure lines under greenhouse conditions, followed by field evaluation (Goulden, 1939).

In the 1940s, it was demonstrated that “shuttle breeding”, or growing a winter generation, could significantly speed up plant breeding. This method involves cultivating two successive generations per year in geographically distinct climatic zones. Beyond expediting the breeding process, its key advantage lies in enabling more stringent selection pressure by evaluating segregating populations across diverse ecological conditions (Borlaug, 2007). The technique gained significant popularity in breeding practice (Ortiz et al., 2007; Collard et al., 2013), despite being limited to producing only two generations annually. For certain countries, geographic constraints necessitate international collaboration with nations capable of cultivating additional generations during autumn-winter periods (Gontcharov et al., 2021).

With the advancement of cellular biotechnology techniques, *in vitro* approaches based on isolated cell and tissue culture have been incorporated into the practice of accelerated pure line development. One such method is embryo culture technology, which involves the isolation of immature embryos 14-20 days after flowering and their cultivation on nutrient media. The regenerated embryos are then transplanted into soil, initiating the growth of a new generation. This approach accelerates breeding by significantly reducing seed maturation time and overcoming postharvest dormancy (Rogo et al., 2023). This method has become fundamental for rapid development of pure lines in numerous economically important plant species (Ochatt et al., 2002; Dağüstü et al., 2012; Liotino et al., 2019). However, the technique presents several limitations, including its labor-intensive nature due to the challenging process of isolating minute-sized embryos, genotype-dependent efficiency, and potential contamination risks during *in vitro* manipulation (Rogo et al., 2023).

Among different methods, the production of doubled haploid (DH) lines has emerged as the most unique and widely demanded approach for generating homozygous plant forms. This technique involves the regeneration of haploid plants followed by chromosome doubling to produce completely homozygous, fertile plants. The average timeframe for obtaining seeds from DH lines ranges from 1 to 1.5 years, depending on the genotype (Germanà, 2011; Watts et al., 2018). Currently, DH production protocols have been established for a vast array of cultivated plant species (Seguí-Simarro et al., 2021). This method is routine and highly effective for a number of crops, including corn (Prasanna et al., 2012; Zararsiz et al., 2019). Despite its remarkable advantages, part of crops have low responsiveness to this method, and therefore researchers continue to address several methodological challenges, including low plant regeneration frequency (Blinkov et al., 2022), albinism (Żur et al., 2021), rooting difficulties (Kozar et al., 2020), and the identification of less toxic chromosome doubling agents (Hooghvorst et al., 2020). These persistent limitations prevent the method from becoming universally applicable and often restrict its large-scale implementation for pure line development.

Among biotechnological methods for rapid development of plants with desired traits, approaches based on transgenic and genome-edited plants have gained prominence (Sidorova et al., 2019; Miroshnichenko et al., 2022). However, the cultivation of such plants remains prohibited by legislation in several countries (Bogatyreva et al., 2021).

Concurrently with advances in biotechnology, the single-seed descent (SSD) method has undergone significant modifications since its inception (Wellensiek, 1962; Fernandez Martinez et al., 1986; Ochatt et al., 2002). These adaptations have included crop-specific optimization of SSD protocols (Fernandez Martinez et al., 1986) and identification of parameters promoting accelerated plant development (Ochatt et al., 2002). Numerous modified SSD protocols were initially based entirely on *in vitro* plant cultivation (Ochatt et al., 2002; Mobini et al., 2015; Yao et al., 2017) or controlled environment growth chambers supplemented with embryo culture for generational turnover (Croser et al., 2016). Over time, due to various limitations, researchers have shifted from *in vitro* cultivation for recombinant line development toward growth chamber-based systems (Mobini and Warkentin, 2016).

The most recently developed approach for rapid generation of pure lines is speed breeding (SB). This concept was inspired by NASA experiments on plant cultivation in enclosed chambers under extended photoperiods in space (Ghosh et al., 2018). SB minimizes the vegetative period of each generation by creating conditions that promote: (1) accelerated flowering, (2) rapid seed maturation, and (3) overcoming postharvest dormancy to enable successive cultivation cycles. The feasibility of this approach for accelerating growth in long-day crops was first demonstrated in 2018. This groundbreaking study achieved six generations per year for spring wheat, barley, chickpea, and pea, along with four generations annually for rapeseed. In practical terms, pure lines of spring cereals and legumes were obtained within one year, while rapeseed required 1.5 years (Watson et al., 2018). A detailed protocol for replicating these results was published concurrently (Ghosh et al., 2018). Notably, although formally published in 2018, the authors had been actively developing and applying this methodology for nearly a decade prior, as evidenced by their earlier works (Hickey et al., 2009, 2010, 2011).

This technology has proven to be simple, cost-effective, and highly adaptable (Watson et al., 2018). SB has been actively integrated into various breeding and research programs worldwide, demonstrating its universal applicability across different growing zones (Hickey et al., 2017; Li et al., 2019; Cha et al., 2020; Vikas et al., 2021). Following its success with long-day crops, optimized protocols were subsequently developed for short-day species, including soybean, amaranth, hemp, and others (Jähne et al., 2020; Schilling et al., 2023). To date, SB protocols have been established for a diverse range of plant species (Chiurugwi et al., 2019), significantly expanding the method’s potential applications in both crop improvement and basic plant research.

To date, numerous comprehensive reviews have been published on SB technology. While some of these reviews primarily focused on SB in one or a group of species (Chiurugwi et al., 2019; Jan et al., 2022; He et al., 2024; Kumar and Walia, 2024), several publications described the potential prospects of SB in genetic and breeding research (Hickey et al., 2019; Bhatta et al., 2021). Additionally, some published reviews briefly summarized all possible applications and achievements of SB (Sharma et al., 2023; Chaudhary and Sandhu, 2024; Imam et al., 2024; Ćeran et al., 2024). However, given the rapid development of new protocols, modifications to existing ones, and the expanding integration of SB into diverse research and applied projects, published reviews quickly become outdated and require continuous updates. Therefore, the aim of this review is to provide a comprehensive systematization of all accumulated factors influencing plant vegetation period reduction throughout the entire history of the method’s existence, to summarize published accelerated growth protocols, and to describe the SB application.

## 2 Factors influencing accelerated flowering, maturation, and overcoming postharvest dormancy

**Figure 2:**
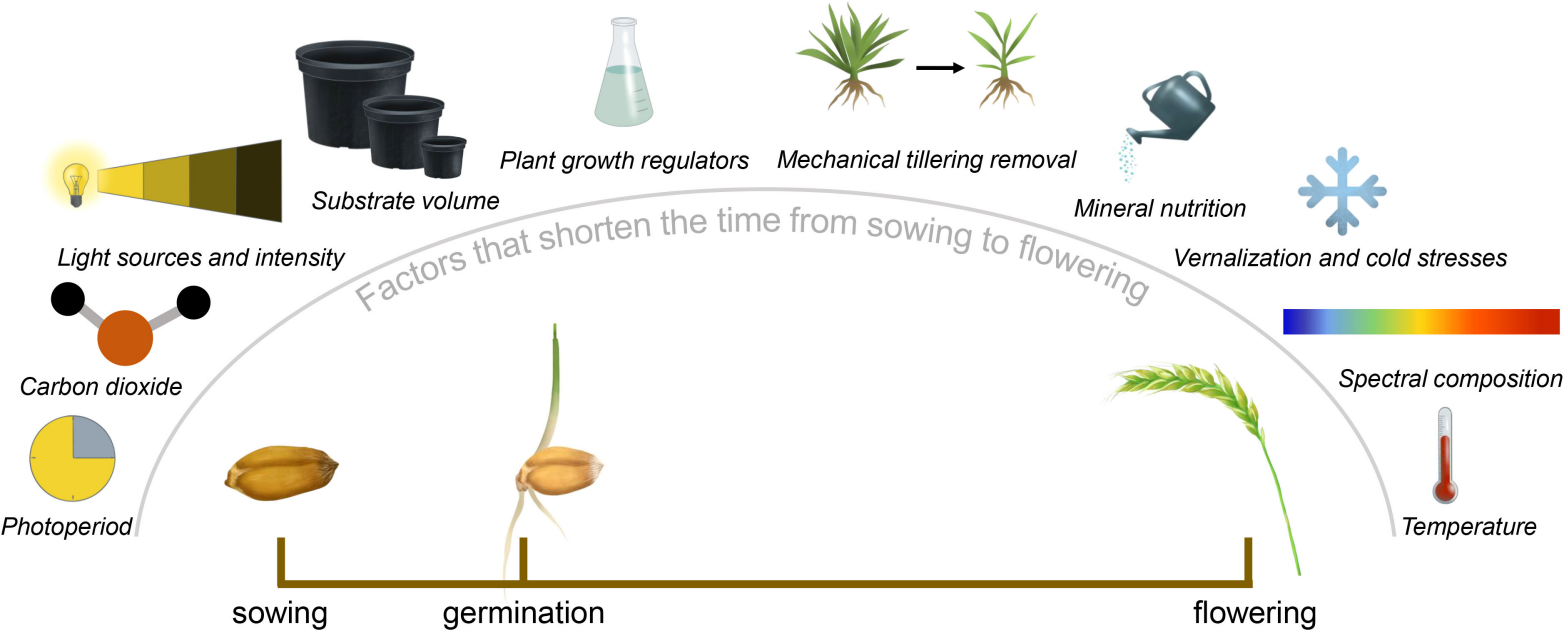
Various factors that shorten the time from sowing to flowering.

**Figure 3:**
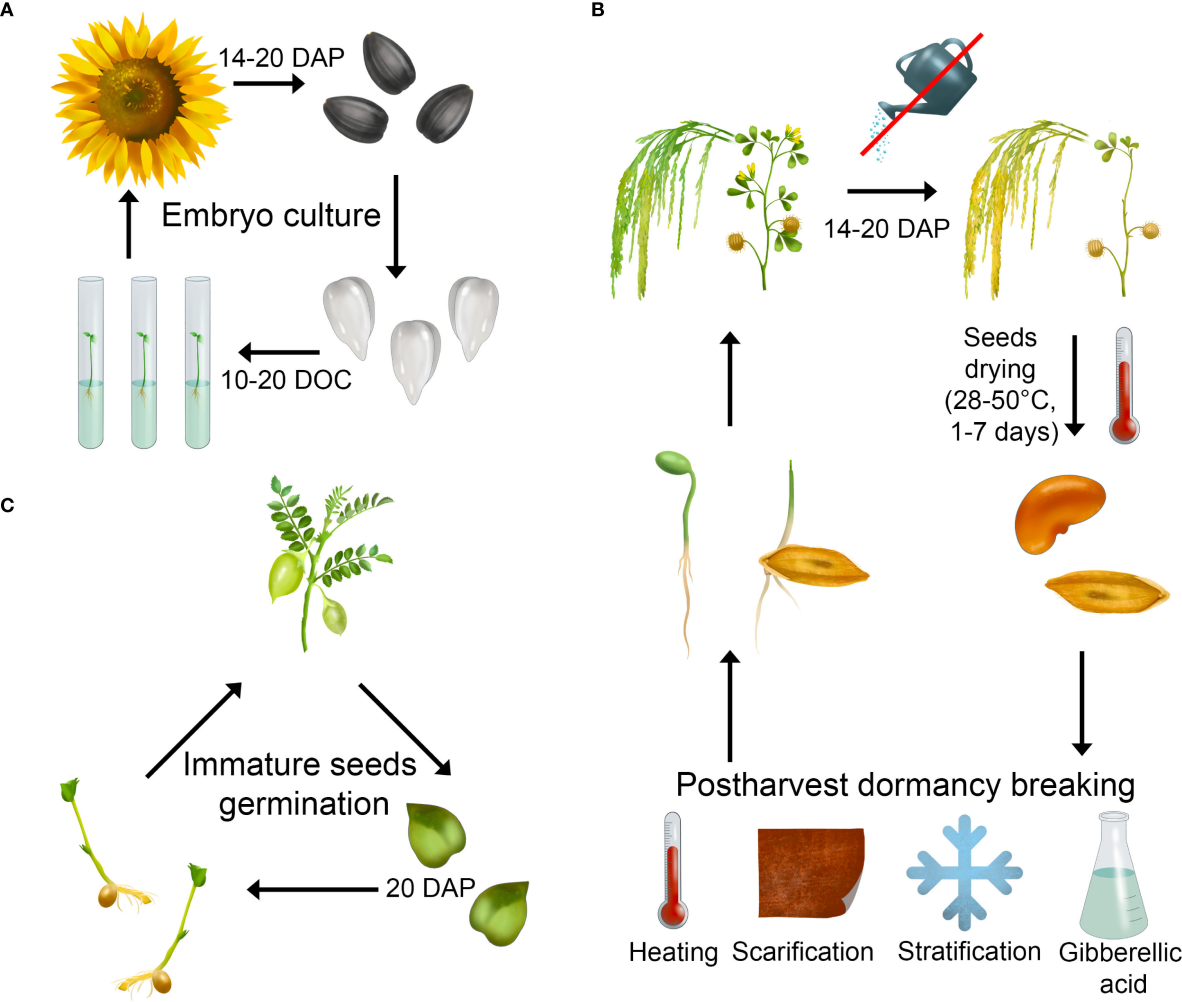
Various approaches to shortening the generative phase of development and overcoming post-harvest seed dormancy: **(A)** embryo culture; **(B)** germination of immature seeds; **(C)** forced drying of immature seeds and exposure to various factors to induce the release from post-harvest dormancy. DAP, days after pollination; DOC, days of cultivation.

All SB protocols are based on previously studied factors that alter plant physiology: accelerated initiation of reproductive organs (**Figure 2**), reduced duration of the generative development phase, and overcoming postharvest dormancy (**Figure 3**). These factors include photoperiod, light spectral composition, temperature, mineral nutrition, among others. The greatest acceleration in development is achieved not by a single factor but by an optimized combination of multiple factors. Moreover, these factors are not universal—parameters that significantly accelerate development in certain crops may have no effect in others.

Currently, SB protocols have been developed for numerous plant families, encompassing both globally significant staple crops and regionally important minor crops. Existing protocols are being progressively modified for large-scale application through the incorporation of parameters that accelerate development, increase throughput, reduce costs, and integrate genotyping (Song et al., 2021; Cha et al., 2023; Sandhu et al., 2024).

Each crop requires specific acceleration approaches primarily determined by its biological characteristics. The developed protocols exhibit genotype-dependent efficacy due to allelic variation in genes controlling growth rate, necessitating the use of cultivars representing different maturity groups during protocol development (Jähne et al., 2020; Harrison et al., 2021; Schoen et al., 2023; Sandhu et al., 2024). Below, we outline the most widely utilized factors that promote accelerated plant development. Understanding these factors and their biological mechanisms enables the development of protocols for new crops and enhances the efficiency of existing ones through targeted modifications.

### 2.1 Photoperiod

Day length serves as a crucial environmental signal for plants, indicating seasonal changes. Unlike annual temperature fluctuations, photoperiodic variation provides a more reliable and consistent indicator of seasonal progression. Plants possess genetically programmed responses to changes in day/night duration, which phenotypically manifest through various developmental processes, including the initiation of reproductive organs, tuber formation, dormancy transition, and other physiological responses (Jackson, 2009).

Based on their photoperiodic response, plants can be classified into three groups: (i) short-day plants initiate flowering when day length falls below a critical threshold (typically 8–15 h of light, depending on the genotype); (ii) long-day plants require day lengths exceeding a critical threshold to flower (typically more than 9-18 hours of light, depending on the genotype); (iii) day-neutral plants exhibit no flowering response to photoperiodic changes. The critical day length represents the specific photoperiod threshold that triggers floral induction when exceeded (for long-day plants) or undershot (for short-day plants). This threshold varies significantly between species and even among cultivars within the same species (Jackson, 2009; Watson et al., 2018; Heikrujam et al., 2022). In SB, optimal day lengths are carefully selected according to a crop’s photoperiodic sensitivity to induce rapid flowering (Ficht et al., 2023; Kigoni et al., 2023; Schilling et al., 2023).

For long-day and day-neutral crops, a photoperiod of 22 hours light/2 hours darkness is recommended. Numerous studies have demonstrated that this extended day length significantly accelerates flowering initiation in Triticeae tribe cereals (Watson et al., 2018; Ficht et al., 2023; Kigoni et al., 2023), pea (Cazzola et al., 2020), rapeseed (Song et al., 2021), and many other species compared to shorter photoperiods. While continuous lighting can be used for long-day crops, incorporating a dark period helps maintain circadian gene expression and improves the physiological status of growing plants (Watson et al., 2018; Choi et al., 2023; Mitache et al., 2023). Artificial lighting need not be employed for the full 22-hour period; in greenhouse conditions, the most economical approach involves utilizing 10 hours of natural daylight supplemented with artificial lighting during dark periods. This combination of natural and artificial lighting results in equivalent developmental rates and seed production per plant compared to extended artificial lighting regimes (Cha et al., 2023).

Short-day crops lack a universal optimal photoperiod, necessitating preliminary evaluation of day length conditions that promote accelerated flowering initiation. Excessively short photoperiods may induce stress responses that delay flowering in these species (Schilling et al., 2023). Research has identified species-specific requirements: pigeon pea demonstrates optimal development under 8 h light/16 h dark cycles (Gangashetty et al., 2024), while rice, soybean, and cowpea achieve maximum flowering acceleration at 10/14h (Jähne et al., 2020; Edet and Ishii, 2022), with hemp and pepper performing best under 12/12h regimes (Liu et al., 2022; Schilling et al., 2023; Somody and Molnár, 2025). Photoperiod manipulation has been successfully implemented for short-day crops through a strategic approach involving initial plant growth under extended photoperiods to promote vigorous biomass accumulation, followed by a switch to shorter day lengths to trigger floral initiation in well-developed plants. The duration of the initial long-day phase represents a critical parameter in this strategy, as improper timing may fail to significantly accelerate flowering upon transition to short-day conditions or, in extreme cases, may even delay the shift from vegetative to reproductive development. Following successful flowering and seed set, subsequent extension of photoperiod can further accelerate seed maturation (Schilling et al., 2023). This photoperiod manipulation technique has demonstrated particular efficacy in several short-day crops including amaranth (Stetter et al., 2016), hemp (Schilling et al., 2023; Somody and Molnár, 2025), pigeon pea (Gangashetty et al., 2024), and rice (Kabade et al., 2024; Sandhu et al., 2024).

As an additional factor, it is recommended to simulate dawn and dusk conditions through gradual increases in light intensity at illumination onset and progressive decreases before complete light termination. While not strictly necessary, this procedure has been shown to enhance the quality of growing plants (Ghosh et al., 2018; Watson et al., 2018). Recommended photoperiods for major crops are presented in **Supplementary Table 1**.

### 2.2 Light sources and spectral composition

Under controlled growth conditions, artificial lighting serves as a substitute for solar radiation, facilitating photosynthesis while influencing photomorphogenesis, photoperiodism, and phototropism. Plant lighting systems encompass incandescent lamps, fluorescent tubes, high-pressure mercury lamps, high-pressure sodium lamps, metal-halide lamps, and light-emitting diodes (LEDs). These lighting sources vary significantly in their spectral characteristics, luminous efficacy, power consumption, operational lifespan, heat emission, reliability, and disposal requirements (Dutta Gupta and Agarwal, 2017).

For SB applications, any lighting systems capable of providing photosynthetically active radiation (PAR) within the 400-700 nm spectrum range are suitable (Ghosh et al., 2018; Watson et al., 2018). The most commonly employed lighting technologies for accelerated plant growth include metal-halide (Ji et al., 2022; Kigoni et al., 2023; Sandhu et al., 2024), high-pressure sodium (Marenkova et al., 2024), fluorescent (Cazzola et al., 2020; Choi et al., 2023; Gimeno-Páez et al., 2025), and LED lighting systems (Cha et al., 2023; Gaoua et al., 2025; Özkan et al., 2025). Hybrid lighting systems combining different lamp types can be implemented to achieve optimal spectral composition (Ghosh et al., 2018; Watson et al., 2018; Zheng et al., 2023). For long-day crops, LED fixtures offer particular advantages due to their energy efficiency during continuous illumination (Watson et al., 2018), with additional benefits including precise spectral tuning capabilities (Jähne et al., 2020; Choi et al., 2023; Zainuddin et al., 2024).

Plants exhibit distinct preferences regarding light spectral composition, necessitating the use of different lighting sources for various crops (Ghosh et al., 2018). For instance, studies on cowpea in SB have demonstrated significantly more successful pollination under LED lighting compared to metal-halide lamps (Edet and Ishii, 2022).

Manipulation of light spectral composition enables modification of key plant growth and developmental parameters (Ouzounis et al., 2015). Far-red light represents one of the most potent morphogenetic factors influencing vegetative growth rate. Plant physiological responses are determined not only by the presence of far-red light but also by its ratio to red light (Demotes-Mainard et al., 2016; Blinkov et al., 2025). In SB, far-red light has proven highly effective for significantly accelerating the transition to reproductive phase in rapeseed (Song et al., 2021), pepper (Choi et al., 2023), triticale (Blinkov et al., 2025), amaranth and rice (Jähne et al., 2020). Depending on the crop species, far-red light can reduce time to flowering by 3-20 days (Jähne et al., 2020; Blinkov et al., 2025). Studies demonstrate that far-red light under SB conditions elevates transcriptional levels of *FLOWERING LOCUS T* (*FT*) homologs, thereby promoting accelerated flowering (Song et al., 2021).

However, not all plants exhibit accelerated flowering under far-red light in SB, including at various red-to-far-red ratios. Crops such as pea (Mobini and Warkentin, 2016) and soybean (Jähne et al., 2020) show no response to this spectrum. Additionally, far-red light may lead to undesirable effects like internode elongation, lodging, and a reduction in the number of grains per spike (Jähne et al., 2020; Choi et al., 2023; Blinkov et al., 2025).

Red and blue light spectra also play significant roles in accelerated plant growth under SB conditions (Ghosh et al., 2018; Jähne et al., 2020; Harrison et al., 2021). Beyond promoting earlier flowering (Harrison et al., 2021) and maturation (Gaoua et al., 2025), these spectra contribute to reduced plant stature and more compact growth habits, which are particularly advantageous for growth chamber cultivation (Jähne et al., 2020; Harrison et al., 2021).

Other light spectra contribute less to flowering acceleration but may influence different growth parameters. For instance, green light has been shown to improve soybean physiological status under SB conditions (Jähne et al., 2020). Not all wavelengths promote vegetation period reduction - the addition of UV-A and near-infrared radiation to the light spectrum has been observed to slow vegetative growth in safflower (Gaoua et al., 2025). Recommended light spectral compositions for SB protocols are presented in **Supplementary Table 1**.

### 2.3 Light intensity

Light intensity represents one of the most critical light quality parameters, alongside spectral composition. Variations in light intensity can affect photosynthetic efficiency and induce changes in plant morphological, anatomical, physiological, and biochemical characteristics (Shafiq et al., 2021). Numerous studies have demonstrated that light intensity also influences the rate of phenological phase progression under SB conditions (Liu et al., 2022; Ficht et al., 2023; Kabade et al., 2024).

Plants require specific light intensity levels sufficient to provide energy for photosynthetic biochemical reactions to ensure efficient growth and development (Shafiq et al., 2021). Optimal light intensity not only facilitates rapid transition to the reproductive phase but also helps maintain plants in good physiological condition (Mitache et al., 2024a). Although plant species exhibit varying light intensity requirements, the most universally applicable intensity in SB ranges between 450-500 μmol/m²/s at adult plant canopy height (Ghosh et al., 2018; Watson et al., 2018; Jähne et al., 2020). Studies on wheat and pepper demonstrate that light intensities below 400 μmol/m²/s result in slower plant development rates (Liu et al., 2022; Ficht et al., 2023).

Increasing light intensity to 800-1000 μmol/m²/s may accelerate flowering and maturation in certain crops such as soybean (Jähne et al., 2020) and rice (Kabade et al., 2024). Higher light intensities can also promote more compact plant architecture, which provides significant advantages in controlled growth environments (Jähne et al., 2020; Mitache et al., 2024a). However, excessively high light intensities may cause photosynthetic photoinhibition, oxidative damage, and deterioration of cellular components, ultimately leading to developmental delays (Shafiq et al., 2021). The negative effects of high light intensities have been experimentally demonstrated in SB for pepper, chickpea, and lentil, manifesting not only as reduced growth rates but also leaf scorching, plant wilting, and poor seed production (Liu et al., 2022; Mitache et al., 2024a). Recommended light intensities for various agricultural crops are presented in **Supplementary Table 1**.

### 2.4 Temperature

Temperature represents a fundamental factor influencing plant development, governing critical processes including seed germination rate, progression through vegetative and reproductive phases, proper meiosis execution, gamete viability, pollination success, and ultimately final yield (Pipattanawong et al., 2009; Hatfield et al., 2011; Fu et al., 2022). Each crop species exhibits specific temperature optima that may vary according to developmental stage (Hatfield et al., 2011). Consequently, SB protocols require careful temperature regime selection tailored to individual crops to maximize developmental rates (Ghosh et al., 2018). The most common daytime temperatures for growing plants under speed breeding conditions are 22-25°C. This daytime temperature range is suitable for most long-day cereals (Watson et al., 2018; Cha et al., 2021; Ficht et al., 2023), members of the *Brassica* genus (Ghosh et al., 2018; Watson et al., 2018; Song et al., 2021) and others (Mobini et al., 2020; Schilling et al., 2023; Mitache et al., 2024a). For warm-season crops, such as rice (Kabade et al., 2024), cotton (Wang et al., 2025), soybean (Nagatoshi and Fujita, 2019), and others (Sajja et al., 2025), daytime temperatures can be increased to 28-32°C. Recommended temperature parameters for major agricultural crops are provided in **Supplementary Table 1**.

Maintaining precise temperature control represents a critical requirement in SB, as significant temperature fluctuations may induce stress responses and reduce vegetative growth rates (Watson et al., 2018; Ficht et al., 2023). However, certain crops such as wheat demonstrate minimal reductions in growth rate or productivity when exposed to temperature variations in greenhouse conditions, including during winter periods, provided other SB parameters remain optimized (Cha et al., 2023). A modest temperature reduction during dark periods, with gradual transitions between light and dark phases, is recommended though not mandatory, as this practice helps mitigate stress during active growth under accelerated development conditions (Watson et al., 2018).

Certain cold stress treatments can be beneficial in SB. For instance, cold hardening at the cotyledon stage accelerates flowering in tomato (Gimeno-Páez et al., 2025), while brief temperature reduction after flowering initiation in faba bean improves seed set in the first lower flowers (Mobini et al., 2020).

### 2.5 Carbon dioxide

Elevated carbon dioxide concentrations significantly influence plant growth, development, biomass accumulation, and yield through their involvement in photosynthesis and secondary carbon metabolism (Gamage et al., 2018). Consequently, supplemental CO_2_ application in accelerated growth chambers may serve as an effective tool for inducing rapid transition to the reproductive phase (Ghosh et al., 2018).

In practice, supplemental CO_2_ does not always accelerate plant flowering. While elevated CO_2_ concentrations significantly hasten rice heading (Tanaka et al., 2016), they show no effect on accelerating phenological phases in cowpea and soybean (Nagatoshi and Fujita, 2019; Edet and Ishii, 2022). Even within a single crop species, additional CO_2_ may shorten the generation period in some genotypes while prolonging it in others, as demonstrated in vegetable soybean (Taku et al., 2024).

Carbon dioxide exerts a more pronounced effect on plant productivity, promoting stem elongation, increased vegetative biomass, greater inflorescence number, improved seed set, and enhanced seed weight (Tanaka et al., 2016; Nagatoshi and Fujita, 2019; Taku et al., 2024). In soybean SB, supplemental CO_2_ increases flower size and number, facilitating emasculation and hybridization procedures (Nagatoshi and Fujita, 2019). The influence of carbon dioxide on productivity demonstrates cultivar-specific responses (Tanaka et al., 2016; Edet and Ishii, 2022).

### 2.6 Vernalization

Vernalization represents a physiological process whereby exposure to low temperatures induces the formation of reproductive organs in winter and biennial crops. This process constitutes an essential requirement for flowering initiation in winter cereals, certain *Brassicaceae* species (including rapeseed and turnip rape), and biennial crops such as sugar beet. The vernalization period represents a significant time constraint in accelerated breeding, requiring up to 70 days depending on genotype, thereby imposing substantial limitations on rapid generation cycling for winter crops (D’Angelo and Goldman, 2019; Song et al., 2021; Cha et al., 2022; Zheng et al., 2023).

An approach to reduce vernalization time in winter cereals was developed, involving vernalization of germinating seeds on the soil surface at 10°C under a 22/2 h day/night photoperiod. This method achieves vernalization within 28 days (Cha et al., 2022). The protocol was further modified by replacing germinating seeds with *in vitro* isolated embryos, which further accelerated the vernalization process (Zheng et al., 2023). However, this protocol demonstrated limited effectiveness and universality: 6 out of 51 tested genotypes failed to flower within 150 days after sowing, while 18 genotypes exhibited spring or facultative growth habits (Schoen et al., 2023). For biennial onion crops, a 12-week vernalization period at 10°C has proven highly effective for floral bud initiation (D’Angelo and Goldman, 2019). Short vernalization periods can also slightly accelerate vegetative growth in spring genotypes, as demonstrated in spring rapeseed (Song et al., 2021).

### 2.7 Mineral nutrition and substrate volume

Mineral nutrition serves as an important flowering inducer, with nitrogen, potassium, and phosphorus having the greatest influence on the rate of phenological phase progression. While plant responsiveness to these elements is highly specific, certain trends exist in fertilizer use for flowering time regulation (Zhang et al., 2022).

Nitrogen, regardless of its form (nitrate or ammonium), promotes active biomass accumulation but delays flowering onset (Zhang et al., 2022). To prevent delayed flowering, nitrogen application should be restricted after the transition to the reproductive stage begins (Kabade et al., 2024).

Potassium and phosphorus are well-established factors promoting accelerated plant flowering. Both excess and deficiency of these elements can induce early flowering - while adequate amounts facilitate faster transition to reproductive phase, their shortage may cause significant stress that also triggers premature flowering (Zhang et al., 2022). Consequently, manipulation of these nutrients has been incorporated into accelerated plant growth protocols to induce early flowering. For instance, rice cultivation employs foliar application of potassium and phosphorus to hasten development (Kabade et al., 2024), whereas tomato production utilizes increased root-zone potassium supplementation to accelerate maturation (Gimeno-Páez et al., 2025).

Micronutrients also significantly influence vegetative growth rate, plant physiological status, and seed quality. So, the use of micronutrients and complex fertilizers application is recommended (Marenkova et al., 2024; Sandhu et al., 2024; Taku et al., 2024; Sajja et al., 2025).

Given the accelerated plant development in SB, continuous monitoring of macro- and micronutrient supply is essential, as nutritional deficiencies may impair physiological status and slow growth rates (Ghosh et al., 2018; Kabade et al., 2024; Marenkova et al., 2024). The method of fertilizer application - either root zone or foliar feeding - also substantially affects vegetative growth and plant physiology in SB (Sandhu et al., 2024). Hydroponic cultivation may be employed for precise control of mineral nutrition and fertilizer regulation in SB (Cazzola et al., 2020).

The substrate volume for plant cultivation also plays a significant role. High-density cultivation in trays with cell sizes up to 100 ml has gained considerable popularity. This approach accelerates flowering initiation by creating stress conditions and inducing competition among plants (Ghosh et al., 2018; Zheng et al., 2023; Sandhu et al., 2024). Furthermore, tray systems enable efficient space utilization in controlled phytotron environments. When using cultivation trays, individual plants typically produce fewer seeds while maintaining germination rates comparable to conventional conditions, making this method ideally compatible with SSD selection (Mobini and Warkentin, 2016; Ghosh et al., 2018; Zheng et al., 2023). Excessively small cell volumes are not recommended as they may lead to substrate desiccation and subsequent growth retardation (Marenkova et al., 2024). Small-cell tray systems have demonstrated particular effectiveness for spring and winter cereals (Ghosh et al., 2018; Zheng et al., 2023), pea (Mobini and Warkentin, 2016), and soybean (Lee et al., 2023). Some researchers employ cultivation trays with cell numbers matching DNA extraction plate formats, facilitating seamless integration of breeding and genetic analyses (Jähne et al., 2020). However, reduced pot size does not always accelerate flowering time. For certain crops such as hemp and tomato, smaller containers actually delay flowering (Schilling et al., 2023; Gimeno-Páez et al., 2025).

Substrate composition also significantly influences the shortening of the vegetative period (Kigoni et al., 2023; Sandhu et al., 2024). For instance, one technique to accelerate flowering involves growing plants in nutrient-poor substrates such as sand (Kigoni et al., 2023). Studies on rice have shown that adding coconut coir delays seed germination (Sandhu et al., 2024). Detailed recommendations for mineral nutrition and substrate volume in SB are provided in **Supplementary Table 1**.

### 2.8 Mechanical tillering removal

This approach has demonstrated effectiveness in accelerating flowering in cultivated cereals. The method involves removing all tillers during plant growth, leaving only the main spike or panicle (Rana et al., 2019; Song et al., 2021; Marenkova et al., 2024). While this technique does not substantially accelerate flowering time, it reduces the duration by up to 5.2 days, depending on the genotype (Tanaka et al., 2016). However, tiller removal promotes more uniform and faster maturation (Marenkova et al., 2024). This method may be suitable for rapid generational turnover but is less effective for accelerated propagation of specific genotypes, as tiller removal reduces both the total seed yield per plant and the number of seeds set in the main spike or panicle (Tanaka et al., 2016).

However, this approach has not gained widespread popularity in accelerated cereal cultivation, as it remains relatively labor-intensive when working with large populations while providing only marginal effects on flowering date (Tanaka et al., 2016).

### 2.9 Plant growth regulators and biologically active substances

Plant growth regulators influence nearly all physiological processes in plants. Synthetic growth regulators have become an integral component of modern agriculture, enhancing rooting efficiency of cuttings, improving crop resistance to biotic and abiotic stresses, thereby affecting yield, and modulating flowering and maturation rates, among other processes (Agudelo-Morales et al., 2021). These compounds have also found application in SB (Ghosh et al., 2018; Mobini et al., 2020; Schilling et al., 2023).

Gibberellic acid (GA) is most commonly used in SB to break seed dormancy (Lulsdorf and Banniza, 2018; Watson et al., 2018; Kabade et al., 2024; Marenkova et al., 2024). Other growth regulators have also proven effective for addressing specific challenges in SB. For instance, flurprimidol, a gibberellin biosynthesis inhibitor, can be used in accelerated pea cultivation to produce compact plants suitable for high-density controlled environments, reducing internode length without compromising yield (Mobini and Warkentin, 2016). Application of 6-benzylaminopurine (6-BAP) enhances pollen germination in faba bean under accelerated growth conditions, promoting earlier seed formation (Mobini et al., 2020). In hemp cultivation, where SB may result in exclusively female flowers complicating self-pollination and hybridization, treatment with silver nitrate and sodium thiosulfate induces male flower development within 14 days in both monoecious and dioecious varieties (Schilling et al., 2023).

### 2.10 Embryo culture, artificial seed drying, and postharvest dormancy breaking

The recommendations outlined in this chapter primarily focus on inducing accelerated flowering initiation, with less emphasis on hastening seed maturation. Achieving full seed maturity remains a prolonged process, compounded by the extended postharvest dormancy periods characteristic of many species. In SB, two principal approaches are employed to reduce maturation time: embryo culture and forced drying of immature seeds.

Embryo culture serves as an effective tool for transitioning to a new growth cycle. This method involves isolating immature embryos from seeds and culturing them on nutrient media. Embryo isolation typically occurs two weeks after flowering. On average, within 10-20 days of culture initiation, the embryos regenerate into plants ready for soil transplantation (**Figure 3A**). This approach proves particularly useful for species with large embryos (Mobini et al., 2020; Rogo et al., 2023). In SB, the method has demonstrated efficacy for crops including pea (Mobini and Warkentin, 2016), cereals (Zheng et al., 2023; Marenkova et al., 2024), tomato (Gimeno-Páez et al., 2025), sunflower (Çil, 2023), and safflower (Gaoua et al., 2025).

Embryo culture proves suitable not only for spring but also for winter crops. Moreover, embryos on nutrient media can undergo vernalization, requiring shorter durations compared to vernalization of germinated seeds. It should be noted that plants regenerated from isolated embryos typically show lower productivity than those grown from seeds (Zheng et al., 2023).

Embryo culture presents several limitations prompting its replacement with more accessible methods. These drawbacks include asynchronous embryo introduction into *in vitro* culture due to varying seed growth rates (Mobini and Warkentin, 2016), the need for specialized equipment and trained personnel (Ghosh et al., 2018), species-specific cultivation requirements (Mobini and Warkentin, 2016; Gimeno-Páez et al., 2025), and risks of embryo contamination with subsequent loss of plant material (Rogo et al., 2023).

The forced drying of immature seeds has emerged as a more efficient alternative to the labor-intensive embryo culture method. The success of this approach largely depends on the drying protocol, particularly seed developmental stage, temperature, and duration of desiccation (Edet and Ishii, 2022). Typically, seeds collected 10-20 days after flowering are used, with watering discontinued several days prior to inflorescence harvest. Drying is conducted in ovens at 28-50°C for 1-7 days (**Figure 3B**) (Ghosh et al., 2018; Edet and Ishii, 2022; Schoen et al., 2023; Bayhan et al., 2024; Sandhu et al., 2024). Silica gel may be employed to accelerate the drying process (González‐Barrios et al., 2021). Alternatively, some protocols utilize *in situ* drying by reducing irrigation and allowing plants to desiccate in pots under greenhouse conditions (Somody et al., 2024). Seeds obtained through forced desiccation exhibit slightly reduced germination rates and delayed sprouting. Moreover, plants derived from artificially dried seeds demonstrate lower productivity compared to those grown from conventionally matured seeds (Ghosh et al., 2018; Watson et al., 2018; Schoen et al., 2023).

The drying method has demonstrated high efficiency and ease of use for cereals (Watson et al., 2018; González‐Barrios et al., 2021; Schoen et al., 2023; Sandhu et al., 2024), legumes (Watson et al., 2018; Nagatoshi and Fujita, 2019; Edet and Ishii, 2022), and cruciferous plants (Watson et al., 2018). The drying protocols for these crops are presented in **Supplementary Table 1**.

Some plant species exhibit postharvest seed dormancy, an adaptive mechanism preventing germination under unfavorable conditions. The duration of dormancy depends on the specific plant genotype and is mediated by genetic factors and exogenous phytohormones, primarily abscisic acid and gibberellins (Finch‐Savage and Leubner‐Metzger, 2006). In SB, various techniques are employed to overcome seed dormancy, including stratification (Watson et al., 2018; Cha et al., 2021; Özkan et al., 2025), scarification (Watson et al., 2018), seed heating (Kabade et al., 2024; Marenkova et al., 2024), GA treatment (Lulsdorf and Banniza, 2018; Kabade et al., 2024; Sandhu et al., 2024), CaCl_2_ solution treatment (Sandhu et al., 2024), or combinations of these methods (Ghosh et al., 2018; Watson et al., 2018; Marenkova et al., 2024). It should be noted that GA application for dormancy breaking may cause negative effects, such as hypocotyl and epicotyl elongation (Jähne et al., 2020). For biennial onions, a unique feature is bulb dormancy after the first growth cycle. Treatment with 15% hydrogen peroxide has proven effective for breaking bulb dormancy in this species (D’Angelo and Goldman, 2019).

In certain crops, seeds enter dormancy only during maturation through gradual accumulation of abscisic acid, while immature seeds remain non-dormant (Finch‐Savage and Leubner‐Metzger, 2006). This principle has been applied in SB for chickpea and soybean, where green but fully formed seeds are used for germination (**Figure 3C**). Plants derived from immature seeds demonstrate normal growth and development (Samineni et al., 2020; Mescouto et al., 2024). However, this method suffers from low germination rates due to fungal infections and rotting of green seeds under the high humidity conditions required for sprouting. Moreover, seedlings from immature seeds exhibit weakened growth, attributable to reduced nutrient reserves in the cotyledons and consequently limited resources for robust seedling establishment (Mescouto et al., 2024). Additionally, plants grown from green mature seeds show increased pod shattering (Samineni et al., 2020).

Seeds should not be sown too deeply after dormancy breaking, as deep sowing delays germination time (Kabade et al., 2024).

### 2.11 Applications of genetic approaches and genetic engineering for speed breeding

Despite the variety of methods described in this chapter for shortening generation time, their application remains limited for certain crops, including sugar beet with its biennial life cycle. To accelerate the development of inbred lines in this species, researchers have demonstrated the potential of incorporating genes promoting rapid flowering. In sugar beet, the *Bd* gene induces early flowering without vernalization under continuous light conditions (24-hour photoperiod). This trait remains inactive under field conditions, preventing premature bolting. Incorporating this gene into SB protocols enables complete plant development within four months (Kroupin et al., 2023; Kuroda et al., 2024).

An alternative approach for accelerating flowering and maturation was demonstrated in tomato through virus-induced overexpression of Arabidopsis *FLOWERING LOCUS T* (*FT*), a key regulator of floral transition. This method involves infiltrating tomato seedling cotyledons at 14 days post-germination with an *Agrobacterium tumefaciens* GV3101 suspension carrying potato virus X vector pGR107 expressing *FT*. The technique does not incorporate foreign DNA sequences into the host genome. Progeny from treated plants show no viral infection symptoms or detectable viral RNA. This approach accelerates flowering and fruit maturation by 14-21 days compared to untreated controls, with the additional benefit of increased flower and fruit production. The method’s efficacy depends on both the viral vector selection and the specific *FT* homolog used, with heterologous *FT* genes proving more effective due to reduced RNA silencing of endogenous genes (Deng et al., 2024).

## 3 Practical application of the speed breeding

**Figure 4:**
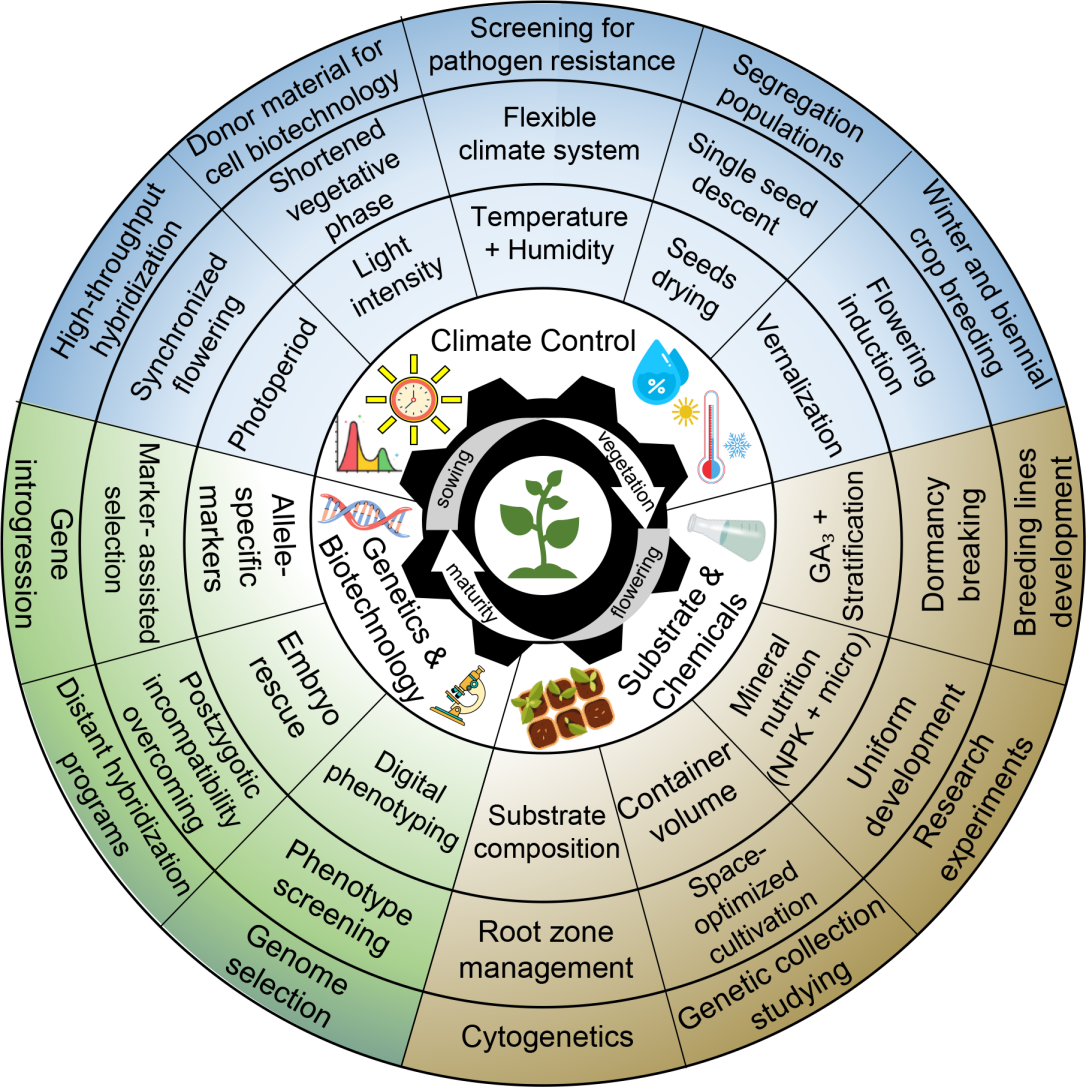
Main speed breeding components and their utilization in plant research.

The advantages of speed breeding (SB), such as rapid plant vegetation, the accelerated transition to the next generation in shorter timeframes, synchronized flowering, the formation of viable seeds, and the simplicity and cost-effectiveness of the developed protocols, make SB a popular and widely used tool in plant breeding, genetics, and other fields of plant research (**Figure 4**). This chapter provides examples of SB applications in various breeding and genetic studies.

### 3.1 Hybridization and segregation of hybrids into lines

SB conditions create a unique opportunity for flowering synchronization both within a single genotype and across multiple genotypes (Watson et al., 2018; Chiurugwi et al., 2019). Synchronization is particularly important for dioecious plants, such as hemp (Schilling et al., 2023). This capability of the SB allows for a large number of crosses, including those in distant hybridization. Under these conditions, seed set is high, and the resulting hybrid seeds exhibit strong germination (Watson et al., 2018; Marenkova et al., 2024). When transitioning from generation to generation under SB conditions, seed germination and the timing of phenological phases remain stable (Watson et al., 2018).

When obtaining F_1_ hybrids, hybridity can be confirmed using molecular markers (Mobini and Warkentin, 2016; Stetter et al., 2016; Edet and Ishii, 2022) or biochemical (protein) markers (Aydin et al., 2024). For DNA extraction in certain crops, a portion of the seed can be used instead of leaves while preserving the embryo. This approach allows for the elimination of non-target material even before sowing (Gangashetty et al., 2024). Phenotypic evaluation is also possible for F_1_ hybrids obtained by crossing a maternal genotype carrying a recessive trait with a paternal genotype carrying a dominant trait (Edet and Ishii, 2022). Assessing hybridity is especially important for plants with small flowers and uncertain hybridization efficiency, such as amaranth (Stetter et al., 2016) and soybean (Nagatoshi and Fujita, 2019).

For segregating hybrids into pure lines under SB conditions, the SSD method is ideally suited. Typically, after hybridization, the next step involves generating a large quantity of F_1_ hybrid seeds in the second growth cycle, followed by sowing all obtained seeds (250–1000 plants) in the third growth cycle and subsequently replanting one seed per plant over 3–4 cycles. This system enables rapid and large-scale line production for both breeding evaluation and genetic studies (Watson et al., 2018; Ficht et al., 2023; Aydin et al., 2024).

When developing recombinant inbred lines for genetic studies, no parallel selection is performed to avoid creating bottleneck conditions (Mobini and Warkentin, 2016). In contrast, for developing breeding lines, selection based on phenotypic traits that show strong correlation between SB and field conditions is possible. In addition to classical phenotyping conducted visually or through manual measurement of various plant parameters, modern digital phenotyping methods can be employed (Alahmad et al., 2018; Watson et al., 2018). Selection and culling of plants in segregating populations can also be performed through genotyping (Watson et al., 2018). To facilitate the assessment of allelic states in segregating populations, it is recommended to sow plants in 96-well trays, which align with 96-well plates used for DNA extraction and PCR. Selection can be based on either a single trait or a combination of traits (Alahmad et al., 2018). However, it should be noted that certain plant traits developed under SB conditions cannot be adequately analyzed without field evaluation. Such parameters include yield and resistance to abiotic stresses. Therefore, breeding lines obtained under SB must undergo final evaluation under field conditions (Watson et al., 2018).

Line development can be conducted entirely under SB, from hybridization to the final generation (Mobini and Warkentin, 2016; Somody and Molnár, 2025), or partially, for example, by accelerating only the early generations (Alahmad et al., 2018) or performing hybridization under conventional conditions while conducting inbreeding under SB (Mitache et al., 2024b; Sandhu et al., 2024). SB can also be utilized during the off-season (Batista et al., 2024).

To date, the rapid development of breeding and recombinant inbred lines under SB has been demonstrated for bread wheat (Hickey et al., 2010, 2012; Cha et al., 2022; Batista et al., 2024), durum wheat (Alahmad et al., 2018), lentil (Lulsdorf and Banniza, 2018; Mitache et al., 2024b), peanut (O’Connor et al., 2013), hemp (Somody and Molnár, 2025), rice (Sandhu et al., 2024), chickpea (Croser et al., 2021), and pea (Mobini and Warkentin, 2016; Cazzola et al., 2020).

### 3.2 Genomic selection

Genomic selection is one of the most promising strategies compared to traditional methods. Today, this approach is becoming increasingly feasible for practical breeding due to recent advances in cost-effective, high-throughput SNP chips and NGS-based platforms for genotyping large segregating populations (Li et al., 2018). The key advantages of genomic selection include its high efficiency in breeding for quantitative traits controlled by multiple loci, each with a minor effect on trait expression (such as yield and resistance to certain diseases). Genomic selection for such quantitative traits offers an additional benefit since these traits are typically evaluated at later stages of the breeding process due to the difficulty of phenotyping (Watson et al., 2019; Pandey et al., 2022; Ćeran et al., 2024; Nannuru et al., 2025). Further advantages of genomic breeding include rapid identification of lines with high breeding value, shortening of the overall breeding cycle, and saving time and resources in cultivar development (Watson et al., 2019; Pandey et al., 2022; Ćeran et al., 2024).

The essence of genomic selection lies in the initial phenotyping and genotyping of a large training population. Genome-wide DNA markers are used for genotyping to assess genomic estimated breeding values (GEBVs) for complex traits. Predictive models are developed based on extensive phenotyping and genotyping data from the training population, which can account for all possible genetic variance for any given trait. Using these predictive models, the breeding value of candidate populations is evaluated, which are also genotyped to assess GEBVs. Lines with the highest breeding value are selected for the next generation (Watson et al., 2019; Pandey et al., 2022; Ćeran et al., 2024).

The greatest efficiency in reducing the breeding cycle time can be achieved by combining genomic selection with SB (Ćeran et al., 2024). Under SB, it is possible to phenotype specific traits in the training population, select plants with high breeding value in candidate populations and create new inbred populations (Watson et al., 2019; Pandey et al., 2022). Experimental results showed that using SB to advance wheat generations from F_2_ to F_8_ can shorten the breeding cycle from 12 to 7 years (Nannuru et al., 2025).

To date, the combined use of genomic selection and SB has demonstrated high efficiency in breeding bread wheat for grain yield (Watson et al., 2019) and resistance to Fusarium head blight (Nannuru et al., 2025). The integration of SB with genomic selection has proven effective in increasing genetic gain compared to conventional phenotypic selection (Nannuru et al., 2025).

### 3.3 Evaluation of plant resistance to pathogens

Numerous experiments demonstrate a strong correlation between plant resistance to various diseases under SB and in the field following artificial inoculation. For example, the disease response to leaf rust observed in wheat plants grown under SB showed a high correlation with field-based measurements (regression analysis, R² = 0.77) (Riaz et al., 2016). Similar results were obtained for fusarium head blight (FHB) and yellow spot (YS) in wheat: phenotypes assessed under SB and field conditions exhibited a strong correlation (Pearson’s correlation was *r* = 0.921 for FHB and *r* = 0.71–0.84 for YS) (Dinglasan et al., 2016; Chhabra et al., 2024). It has been observed that disease symptoms in susceptible plants are more pronounced under SB than in field conditions, facilitating easier evaluation and phenotyping (Chhabra et al., 2024). This occurs because inoculation efficiency in the field often depends on weather conditions, whereas SB allows controlled temperature and humidity regimes that promote better pathogen growth (Hickey et al., 2012). Additionally, mycotoxin accumulation in grains is more active under SB compared to field-grown plants (Chhabra et al., 2024). A particular advantage is that SB enables up to six consecutive resistance evaluations per year, compared to just one in the field (Riaz et al., 2016).

Beyond assessing resistance to a single disease, it is possible to select for complex disease resistance, which helps to identify fewer than 5% of resistant plants in a segregating population. This requires repeated inoculation of test plants during accelerated growth with a pathogen complex throughout the vegetative period (Hickey et al., 2017). Inoculation should be performed at the specific developmental stage when infection by a particular pathogen occurs. Inoculating plants at non-characteristic stages may yield false resistance or susceptibility results (Hickey et al., 2012). For more accurate evaluation, it is recommended to use not just one but a set of the most virulent strains of a given pathogen (Hickey et al., 2017).

Optimal conditions are crucial for active pathogen growth during artificial inoculation. Several studies have shown that extended photoperiods and temperatures maintained at 22°C day/18°C night, do not inhibit pathogen growth. However, for better infection rates under artificial inoculation in SB, lowering temperatures and increasing humidity to 100% is recommended. Improper environmental conditions may produce false resistance indicators (Hickey et al., 2012, 2017).

When selecting plants from segregating populations under SB with artificial disease pressure, the resulting lines demonstrate comparable resistance in field conditions. Moreover, this selection method is more precise than molecular marker-assisted selection, as not all resistance genes are currently known. However, with well-validated molecular markers, parallel selection based on resistance gene presence and artificial disease pressure would be ideal, enabling more accurate dual evaluation (Hickey et al., 2012, 2017).

For more precise selection of resistant and susceptible plants to specific pathogens, preliminary work is necessary to evaluate reference varieties with known resistance and establish correlations between infection rates in SB and field conditions. Additionally, each evaluation of segregating populations should include control varieties with known resistance to avoid false results caused by improper growing or inoculation conditions (Hickey et al., 2011, 2012, 2017).

Published studies listed in **Table 1** demonstrate that accelerated plant growth platforms with parallel pathogen resistance evaluation can serve as an excellent alternative for rapid selection of highly resistant genotypes from segregating populations in immunity breeding, as well as for screening germplasm collections for resistance genes against specific pathogens.

**Table 1:** List of crops and diseases with their pathogens for which selection can be conducted under speed breeding conditions.

Crop species	Diseases and Pathogens	References
Common wheat (*T. aestivum* L.)	Yellow rust (*Puccinia striiformis* f. sp. *tritici* Westend.)	(Hickey et al., 2012)
	Tan spot (*Pyrenophora tritici-repentis* (Died.) Drechsler)	(Dinglasan et al., 2016)
	Leaf rust (*Puccinia triticina* f. sp. *tritici* Erikss.)	(Riaz et al., 2016)
	Fusarium head blight (*Fusarium graminearum* Schwabe)	(Zakieh et al., 2021)
		(Chhabra et al., 2024)
Durum wheat (*T. durum* Desf.)	Crown rot (*Fusarium pseudograminearum* O’Donnell & T. Aoki)	(Alahmad et al., 2018)
	Leaf rust (*Puccinia triticina* Erikss.)	
Barley *(H. vulgare L.)*	Dwarf rust (*Puccinia hordei* G.H.Otth)	(Hickey et al., 2011)
	Leaf rust (*Puccinia hordei* G.H.Otth)	(Hickey et al., 2017)
	Spot form net blotch (*Pyrenophora teres* f. sp. *maculata*)	
	Net form net blotch (*Pyrenophora teres* f. *teres*)	
	Spot blotch (*Cochliobolus sativus*)	
Lentil (*Lens culinaris* Medik.)	Root rot (*Aphanomyces euteiches* Drechs.)	(Lulsdorf and Banniza, 2018)

### 3.4 Gene introgression

SB has gained particular popularity for rapid gene introgression into existing commercial varieties and hybrid parent lines. Near-isogenic lines are developed under SB through successive backcrossing (Cha et al., 2024). Gene pyramiding is achieved by crossing near-isogenic lines of the same variety carrying different loci of a target trait (Baloch et al., 2024). Since accelerated growth technology is highly compatible with marker-assisted selection, it becomes possible to quickly eliminate plants in segregating populations that do not carry the desired allele (Watson et al., 2018; Rana et al., 2019; Song et al., 2021; Cha et al., 2024; Taku et al., 2025). Moreover, due to the high correlation between SB and field conditions for many traits, phenotypic selection can be conducted alongside molecular marker use (Hickey et al., 2009, 2017; Alahmad et al., 2018). In addition to transferring single alleles (Cha et al., 2024), SB also enables haplotype introgression (Song et al., 2021), gene pyramiding (Alahmad et al., 2018; Baloch et al., 2024), and allele replacement (Taku et al., 2025).

To confirm the introgression of a specific gene into a commercial variety or parent line, in addition to marker-assisted verification, the resulting backcrosses are either resequenced or evaluated for similarity to the recurrent parent using KASP or SSR markers (Rana et al., 2019; Song et al., 2021; Cha et al., 2024; Taku et al., 2025; Wang et al., 2025). Selecting plants with the highest genomic similarity to the original parent (over 90%) allows the development of isogenic lines where the impact on key agronomic traits is minimized. This enables more precise study of trait expression in modified lines while preserving the original variety’s potential during selection (Rana et al., 2019; Cha et al., 2024). Molecular markers for assessing recurrent parent genome recovery reduce the need for excessive backcrossing, allowing selection to stop once a high percentage of recurrent parent genome is restored (Taku et al., 2025).

The effectiveness of SB for gene introgression has been demonstrated in improving wheat gluten quality by introgression allele variants of *Glu-B1* (Aydin et al., 2024; Cha et al., 2024), developing salt-tolerant rice lines by introgression *hst1* gene (Rana et al., 2019), creating high-yielding and clubroot-resistant rapeseed by introgression of *BnaA9.CYP78A9a* haplotype and *CRA3.7*, *CRA08.1* and *CRA3.2* loci respectively (Song et al., 2021; Baloch et al., 2024), direct improvement of fiber fineness and fiber yield of cotton by introgression *iaaM* gene (Wang et al., 2025) and producing vegetable soybean with disrupted lipoxygenase-2 synthesis to eliminate beany flavor by replacing allele *Lox2* with an alternative *lox2* allele (Taku et al., 2025).

### 3.5 Evaluation of phenotypic expression of agronomically valuable traits

In the SB, which primarily feature environmental parameters atypical of field conditions, selection based on most phenotypic traits cannot be conducted as in conventional field breeding. For instance, experimental evidence shows no correlation between field and SB for traits such as wheat awn length and spikelet number per spike (Cha et al., 2023). However, certain traits expressed under SB demonstrate high correlation with field performance, enabling effective selection within the accelerated growth system. Moreover, since SB maintains stable growing conditions across generations, plant trait expression remains consistent and unaffected by meteorological variability (Watson et al., 2018).

To determine the feasibility of trait selection under SB, preliminary experiments are conducted using contrast collections that exhibit differential expression of target traits. These studies compare phenotypic performance under SB with multi-year field observations (Hickey et al., 2009; Watson et al., 2018). The validated traits demonstrating high field-SB correlation and thus suitable for phenotypic selection are presented in **Table 2**.

**Table 2:** Phenotypic traits exhibiting high correlation in their expression under speed breeding and field conditions.

Crop species	Trait	References
Wheat	Pre-harvest sprouting	(Hickey et al., 2009)
	Plant height	(Alahmad et al., 2018; Watson et al., 2018; Özkan et al., 2022; Cha et al., 2023)
	Growth rate	(Watson et al., 2018; Özkan et al., 2022; Cha et al., 2023)
	Spike length	(Cha et al., 2023)
	Presence of awns	(Watson et al., 2018)
	Angle of seminal roots during germination	(Richard et al., 2015)
	Number of seminal roots	(Richard et al., 2015)
Barley	Presence of leaf wax coating	(Watson et al., 2018)
Rapeseed	Pod shattering	(Watson et al., 2018)
Hemp	Stem color	(Somody and Molnár, 2025)

### 3.6 Plant genetics research

The capabilities of SB enable rapid evaluation of plant genetic bank collections and mutant collections under laboratory conditions for multiple traits, described above (Hickey et al., 2009, 2012; Watson et al., 2018; Cha et al., 2023) including growth period duration, plant height (Watson et al., 2018; Cha et al., 2023), morphological characteristics (Watson et al., 2018), disease resistance (Hickey et al., 2012, 2017), and product quality (Hickey et al., 2009). Such screening can be conducted over several consecutive growth cycles to confirm trait expression. Rapid analysis of plant collections under SB facilitates the selection of donors for economically valuable traits, as well as the identification of new allelic variants for integration into breeding programs (Li et al., 2018).

SB also enables the development of recombinant inbred lines for gene mapping and marker identification (Christopher et al., 2015; Mobini and Warkentin, 2016; Sandhu et al., 2024). Furthermore, these lines can be directly mapped under SB (Hickey et al., 2011). Additionally, SB allows rapid fixation of traits controlled by monogenic inheritance in a homozygous state (Somody et al., 2024).

The rapid introgression of target alleles using SB and marker-assisted selection facilitates the creation of near-isogenic lines for gene effect studies. Moreover, by assessing the degree of recurrent parent genome recovery, it becomes possible to quickly develop true near-isogenic lines, where the effect of introgressed alleles is not confounded by residual donor genome segments (Song et al., 2021; Cha et al., 2024; Taku et al., 2025).

SB allows manipulation of lighting and growth conditions, significantly influencing vegetative growth rates and trait expression. These conditions are suitable for GWAS analysis to study quantitative trait loci related to flowering time and vegetative period duration, as well as for identifying candidate genes associated with these traits (Choi et al., 2023; Rossi et al., 2024).

### 3.7 Cytogenetics research

One area of cytogenetics research involves studying chromosome behavior during meiosis. Investigating meiotic characteristics requires young buds containing immature pollen (Alexandrov et al., 2022). SB proves useful for rapid cytogenetic studies of meiosis, as it enables quick production of plant material in the form of young buds.

Currently, few cytogenetic studies have utilized SB for growing plant research material. However, studies on wheat and wheat-rye hybrids contrasting for the *Ph1* (*PAIRING HOMOEOLOGOUS 1*) locus, grown under both SB and conventional conditions, revealed no significant differences in chromosome pairing and recombination in meiocytes during metaphase I. Chromosome behavior suggests that both wheat and wheat-rye hybrids are cytologically stable under SB (Watson et al., 2018).

SB facilitates distant hybridization, allowing tracking of introgression segment size and frequency in subsequent segregating generations using FISH analysis. In such cases, karyotyping is conveniently performed on selected root tips from plants grown in trays (Li et al., 2024).

### 3.8 Plant biotechnology

For developing transgenic or gene-edited plants in certain crops, donor explant material is necessary. In most cereals, immature embryos serve as the optimal explant for transformation (Ishida et al., 2007; Shimizu-Sato et al., 2020; Miroshnichenko et al., 2021). SB can accelerate the production of donor explant material, proving valuable for plant biotechnology research. Current studies demonstrate that barley embryos developed under SB conditions exhibit high morphogenetic potential, with transformation efficiency comparable to conventionally grown plants (Watson et al., 2018).

An additional advantage lies in the rapid cultivation of T_0_ plants, enabling quick seed production from transgenic or edited plants. Subsequent sowing of segregating T_1_ populations allows evaluation for homozygous/hemizygous insertions or their absence. Homozygous transgenic or edited plants can be phenotypically characterized under SB conditions while assessing impacts on key agronomic traits (Watson et al., 2018; Hatta et al., 2021).

SB also facilitates distant hybridization. While postzygotic incompatibility may cause embryo abortion in such crosses, SB promote viable embryo formation suitable for embryo rescue techniques, thereby expanding genetic diversity (Li et al., 2024).

### 3.9 Other research experiments

Accelerated plant development in SB enables rapid assessment of growth responses to various factors. By manipulating light intensity (Liu et al., 2022), spectral composition (Jähne et al., 2020), and photoperiod (Schilling et al., 2023), researchers can investigate plant photobiology. Agrochemistry experiments efficiently evaluate cultivar requirements for macro- and micronutrients while determining their effects on final yield (Sandhu et al., 2024). Furthermore, customized growth conditions permit accelerated studies of physiological processes such as pigment biosynthesis during fruit ripening (Ma et al., 2024).

## 4 Prospects and limitations of speed breeding

Currently, speed breeding (SB) protocols have been developed and actively used for applied and fundamental research in only a limited number of crops. To facilitate and improve research activities, as well as cultivar and hybrid development, it is necessary to establish accelerated growth protocols for all economically important plant species. Numerous studies on various crops demonstrate the influence of different conditions on plant growth rates (Croser et al., 2016; Pazos-Navarro et al., 2017), which could serve as a foundation for developing new SB protocols for additional crops.

For perennial and woody species, this method may face significant limitations and low cost-effectiveness. However, several techniques to accelerate flowering in perennial plants have been developed, including grafting onto early-flowering rootstocks (Ceballos et al., 2017), creating optimal growth conditions in greenhouses with plant growth regulators (Aldwinckle, 1975), and applying stratification and embryo culture to overcome seed dormancy (Van Nocker and Gardiner, 2014). These and other approaches could form the basis for developing SB protocols for woody and perennial species. In biennial crops (such as root crops – carrots, turnips, and beets), positive selection for flowering is possible since genotypes more prone to flowering after vernalization will gain a competitive advantage (Kroupin et al., 2023).

Breeding new plant varieties and hybrids for end-product quality is an extremely challenging task. Typically, quality analysis requires large amounts of plant material in the form of seeds, flour, or vegetative biomass. In the early stages of the breeding process (F_1_-F_4_), due to high material heterogeneity and limited output from individual samples, no quality assessment is performed. All quality evaluations are conducted exclusively in later breeding cycles (F_5_-F_7_), which demands additional investment. The integration of speed breeding with CRISPR/Cas editing (Miroshnichenko et al., 2024) and/or marker-assisted selection (Nakamura et al., 2002; Divashuk et al., 2011) opens new possibilities for rapidly developing improved varieties and hybrids with targeted quality traits. This approach is already being actively used in speed breeding programs to develop wheat lines with either enhanced baking qualities (Aydin et al., 2024; Cha et al., 2024) or grain starch properties optimized for advanced deep processing (Alkubesi et al., 2025).

Modification of existing protocols remains relevant, with a priority focus on identifying factors that could further reduce the vegetative period. Currently, the time required to grow one generation under SB protocols ranges from several months (Kuroda et al., 2024) to one year (D’Angelo and Goldman, 2019) for certain crops. Among potential physiological factors not yet utilized in SB protocols, drought stress (Anjum et al., 2017), for example, could promote accelerated plant development. A key focus in optimizing SB protocols is developing conditions that maintain optimal plant physiology under stress, thereby enhancing seed yield and quality over multiple breeding cycles. Additionally, a key aspect of modifying existing protocols involves identifying factors that reduce genotype dependence while overcoming technical challenges, such as developing alternative embryo culture methods for certain protocols.

Developing more accelerated growth protocols would reduce reliance on model organisms for research and shift focus to economically valuable crop species. One advantage of Arabidopsis over wheat, for instance, is its rapid life cycle, whereas SB can shorten wheat’s vegetative period to two months, comparable to Arabidopsis (Watson et al., 2018).

However, SB cannot replace field-based breeding or line testing, as controlled growth conditions in SB differ significantly from conventional field conditions, for which these lines are ultimately developed. Nevertheless, numerous positive correlations have already been identified for traits such as disease resistance (Hickey et al., 2012, 2017), plant height (Watson et al., 2018; Cha et al., 2023), and morphological characteristics (Watson et al., 2018) between SB and field conditions. Identifying additional correlations and testing them across other crops would enable direct selection under artificial growth conditions and culling within segregating SB populations. However, it is challenging to identify correlations in the selection of traits for productivity, as well as resistance to abiotic and various biotic factors.

Despite this, SB cannot replicate field conditions for phenotypic evaluation of certain traits (Somody and Molnár, 2025). Due to the relatively short existence of this method, its current active application is primarily limited to approaches such as developing pure breeding or recombinant inbred lines (Batista et al., 2024; Mitache et al., 2024b; Somody and Molnár, 2025), as well as gene introgression (Song et al., 2021; Cha et al., 2024; Taku et al., 2025). In the future, this method may find broader application in other applied and fundamental research areas. For instance, the SB represents an ideal platform for training students in skills such as conducting hybridization, developing breeding lines, their evaluation and selection, as well as creating mapping populations. Modern educational programs, including bachelor’s, master’s, specialist, and postgraduate degrees, typically last only 2-4 years. This timeframe is insufficient for conventional methods to not only develop pure lines but also conduct additional research with them (Bhatta et al., 2021; Wanga et al., 2021).

Another potential application of SB is the accelerated evaluation of developed cultivars and hybrids to expedite their registration and enable a faster replacement pipeline. Currently, the process of evaluating new plant hybrids and cultivars for distinctness, uniformity, stability, and novelty (DUS) takes approximately 3 years. Uniformity and stability can theoretically be assessed under SB by evaluating morphological traits such as the presence of awns, growth rate, plant height, etc., over two consecutive generations. After this evaluation, only those cultivars and hybrids that show no segregation under SB would be selected for field testing. This approach could significantly streamline the registration of new breeding achievements (Jamali et al., 2020).

The SB, including growth chambers with controlled lighting, temperature, CO_2_, etc., can be costly. Moreover, extended use of artificial lighting may lead to additional electricity costs. The economic aspect presents a potential limitation to the widespread adoption of SB. However, investments in such chambers may be quickly recouped through rapid development of new breeding material (Pandey et al., 2022; Nannuru et al., 2025). Several registered cultivars of major agricultural crops developed using SB have already been released (Chaudhary and Sandhu, 2024), and this list will undoubtedly expand in the near future.

## 5 Conclusion

Speed breeding is a relatively recent technique that has gained widespread recognition and interest among scientists, primarily due to its simplicity since the protocols rely on controlled factors such as photoperiod, light source characteristics (including intensity and spectral composition), temperature regimes, substrate volume, application of plant growth regulators, carbon dioxide supplementation, mechanical tiller removal, optimized mineral nutrition, as well as vernalization and cold stress treatments. This technology has proven to be simple, cost-effective, and highly adaptable. The method’s versatility has enabled its application across diverse research areas including hybridization and subsequent segregation of hybrids into pure lines, genomic selection procedures, assessment of plant resistance to pathogens, targeted gene introgression, evaluation of phenotypic expression in agronomically important traits, fundamental studies in plant genetics and cytogenetics, biotechnological applications, and various specialized experiments ranging from photobiological research to agrochemical testing and physiological investigations.
