# Correction: Speed breeding: protocols, application and achievements

**DOI:** 10.3389/fpls.2025.1740157

**Published:** 2025-11-17

**Authors:** Andrey Olegovich Blinkov, Pavel Yuryevich Kroupin, Anna Ruslanovna Dmitrieva, Alina Alexandrovna Kocheshkova, Gennady Ilyich Karlov, Mikhail Georgievich Divashuk

**Affiliations:** Department of Applied Genetic Technologies, All-Russia Research Institute of Agricultural Biotechnology, Moscow, Russia

**Keywords:** speed breeding, pure lines, accelerated flowering, breeding, homozygous lines, dormancy breaking

An incorrect number was provided for the **Funding** statement, “Agreement No 075-15-2025075-15–581 dated June 23, 2025”. The correct number is “Agreement No 075-15-2025–581 dated June 23, 2025”.

The original version of this article has been updated.

