# Supplementary material for: Speed breeding: protocols, application and achievements
Source: Front Plant Sci. 2025 Sep 17;16:1680955. doi: 10.3389/fpls.2025.1680955 (PMC12484230; doi:10.3389/fpls.2025.1680955)
Supplement: Supplementary file 1 [file Table1.docx]

**Supplementary Table 1.** Developed protocols of Speed Breeding for general crops and model plants species. The flowering time, duration of one generation, and number of generations per year may differ among genotypes and are presented as a range of values

| **Crop species** | **Growing conditions for accelerated flowering, ripening and overcoming post-harvest seed dormancy** | **The flowering time and (if necessary) duration of vernaliztion** | **Generation cycle duration (days); generations per year** | **References** |
| --- | --- | --- | --- | --- |
| ***Amaranthaceae*** | | | | |
| Amaranth  (*Amaranthus* spp.) | **Growing:** 7×7 cm pots. Initial two weeks under 16/8 h day/night photoperiod at 35/30°C day/night, followed by 8/16 h day/night photoperiod at 30/25°C day/night. Light intensity 150 μmol/m²/s | Flowering at 4 weeks | 2 months; 6 generations | (Stetter et al., 2016) |
|  | **Growing:** 10/14 h day/night photoperiod; light intensity 556 μmol/m²/s; spectrum enriched with far-red light | Flowering at 35 days | No data on generation time and generations per year | (Jähne et al., 2020) |
| Quinoa  (*Chenopodium quinoa* Willd.) | **Growing:** 22/2 h day/night photoperiod with 1 h sunrise/sunset simulation; light intensity 440-650 μmol/m²/s at adult plant level. 22/17°C day/night, gradual changes during sunrises/sunsets. Humidity 70% | Flowering at 54.6 days | 78.6 days; 4.5 generations | (Ghosh et al., 2018) |
| Sugar beet  (*Beta vulgaris* L.) | **Growing:** Plants carrying *Bd* gene grown under 24 h daylength with temperature not below 15°C | Bolting at 35-55 days | 4 months; up to 3 generations | (Kuroda et al., 2024) |
| ***Amaryllidaceae*** | | | | |
| Onion  (*Allium cepa* L.) | **First growing cycle:** field conditions with conventional сrop establishment, bulbs dug up in autumn  **Second growing cycle:** Bulbs treated with 15% hydrogen peroxide for 4 hours for dormancy breaking, then grown at 20°C under 16/8 h day/night photoperiod for three weeks. Then, **vernalization** at 10°C for 12 weeks with 16/8 h day/night photoperiod. After vernalization **growing** at 20°C with 16/8 h day/night photoperiod | Complete growth cycle less than 12 months | | (D’Angelo and Goldman, 2019) |
| ***Asteraceae*** | | | | |
| Safflower  (*Carthamus*  *tinctorius* L.) | **Growing:** 22 h day/2 h night photoperiod; LED lighting with red:blue:white spectrum (1:1:1 ratio); light intensity 1018 μmol/m²/s. 26°C. Humidity 70%  **Generation turnover:** embryos isolating at 20-25 days after flowering | Flowering at 22,6 days | 50.6 days; 6 generations | (Gaoua et al., 2025) |
| Sunflower  (*Helianthus annuus* L.) | **Growing:** 22/2 h day/night photoperiod; LED lighting. 24/18°C day/night. Humidity 70%  **Generation turnover:** embryo isolation at 13 days after flowering. Petri dishes cultured under 22/2 h day/night photoperiod at 25°C | Flowering at 52-67 days | embryo isolation at 65-80 days; 4-5 generations | (Çil, 2023) |
| ***Brassicaceae*** | | | | |
| Cabbage  *Brassica oleracea* L. | **Growing:** 22/2 h day/night photoperiod with 1 h sunrise/sunset simulation; light intensity 440-650 μmol/m²/s at adult plant level. 22/17°C day/night, gradual temperature changes during sunrises/sunsets. Humidity 70% | Flowering at 49.2±1.8 days | 155 days; up to 2 generations | (Ghosh et al., 2018) |
| Rapeseed (spring)  (*Brassica napus* L.) | **Growing:** 22 h day/2 h night photoperiod with 1 h sunrise/sunset simulation; light intensity 440-650 μmol/m²/s at adult plant level. 22/17°C day/night, gradual temperature changes during sunrises/sunsets. Humidity 70%  **Generation turnover:** pods collected 6 weeks after sowing and dried at 35°C for 5 days. Seeds placed on moist filter paper in agar plates overnight at room temperature, then stratified at 4°C for 4 days followed by 5 days at room temperature | Flowering at 44-49 days | 75-80 days;  4 generations | (Watson et al., 2018) |
| Rapeseed (winter)  (*Brassica napus* L.) | **Vernalization:** germinating seeds. 22/2 h day/night photoperiod. 4.5/9°C day/night. Duration depends on genotype  **Growing:** 22/2 h day/night photoperiod; LED full-spectrum lighting (emphasize on blue, red and far-red); light intensity >900 μmol/m²/s at 10 cm from light source. 22°C. Humidity 70% | vernalization 17-55 days, bud formation at 42-92 days | 87-125 days; up to 4 generations | (Song et al., 2021) |
| Turnip  *Brassica rapa* L. | **Growing:** 22/2 h day/night photoperiod with 1 h sunrise/sunset simulation; light intensity 440-650 μmol/m²/s at adult plant level. 22/17°C day/night, gradual temperature changes during sunrises/sunsets. Humidity 70% | Flowering at 36.5±2.5 days | 112 days; up to 3 generations | (Ghosh et al., 2018) |
| ***Cannabaceae*** | | | | |
| Hemp  (*Cannabis sativa* L.) | **Growing:** 1 L pots. Continuous lighting for first two weeks, then switched to 12/12 h day/night photoperiod for 4 weeks (or until first seeds appear), followed by continuous lighting. LED lighting with 500 μmol/m²/s intensity at 15 cm from light source. 25°C. For male flower induction, plants treated with 1.5 mM silver nitrate and 6 mM sodium thiosulfate. Treatment repeated 3-5 times at 3-day intervals  **Maturation:** irrigation stopped after seed formation | Flowering at 29.2-33.3 days | 61 days; up to 6 generations | (Schilling et al., 2023) |
|  | **Growing:** greenhouse conditions with initial photoperiod 16/8 h day/night, after several weeks, photoperiod changed to 12/12 h day/night. Humidity 50-70%. 21°C  **Maturation:** irrigation stopped after seed formation | Flowering at 45-50 days | 79-92 days; 5 generations | (Somody et al., 2024; Somody and Molnár, 2025) |
| ***Fabaceae*** | | | | |
| Alfalfa  (*Medicago truncatula* Gaertn.) | **Growing:** 1 L pots. 22/2 h day/night photoperiod with 1 h sunrise/sunset simulation; light intensity 360-380 μmol/m²/s at shelf level. 22/17°C day/night, gradual temperature changes during sunrises/sunsets. Humidity 70%  **Generation turnover:** irrigation stopped when 90% well-formed pods appear. Pods collected after 14 days. Seeds scarified with sandpaper, sterilized, soaked for 2 h and then transferred to agar plates for 72 h at 4°C | Flowering at 35.3±0.5 days | 89.0±1.4 days; 4 generations | (Watson et al., 2018) |
| Chickpea  (*Cicer arietinum* L.) | **Growing:** 1.4 L pots. 22/2 h day/night photoperiod with 1 h sunrise/sunset simulation; light intensity 440-650 μmol/m²/s at adult plant level. 22/17°C day/night with gradual changes during sunrises/sunsets. Humidity 70%. Weekly foliar feeding with calcium nitrate (1 g/L)  **Maturation:** irrigation is gradually stopped one week before harvest. Cut pods are dried at 35°C for 5 days. Post-harvest dormancy is overcome by incubating seeds on moist filter paper in Petri dishes with agar overnight at room temperature for imbibition, followed by 4 days at 4°C. Seeds are then germinated for 5 days at room temperature | Flowering at 28.6–31.4 days | 60 days;  4-6 generations | (Watson et al., 2018) |
|  | **Growing:** semi-controlled greenhouse conditions. Supplemental nighttime lighting to extend photoperiod  **Generation turnover:** germination of immature seeds in Petri dishes at 21-23 days after flowering | Flowering at 23–56 days | 43-79 days; 6-7 generations | (Samineni et al., 2020) |
|  | **Growing:** 18/6 h day/night photoperiod with light spectrum 410-730 nm and light intensity 74-93 μmol/m²/s (4000-5000 lux). 24/15°C day/night | Flowering at 28 days | 54 days; 6 generations | (Mitache et al., 2024a) |
| Edamame (Vegetable soybeans)  (*Glycine max* (L.) Merrill) | **Growing:** 10/14 h day/night photoperiod; light intensity 220 μmol/m²/s at plant level. 30/25°C day/night. CO₂ level maintained at 550 ppm. Humidity 70-80%  **Generation turnover:** pods harvested when color changes from green to yellow (R7 developmental stage), dried for 7 days at 25±1°C and 10-20% humidity | Flowering at 28,4-39,4 days | 74-85 days, 4-4.5 generations | (Taku et al., 2024, 2025) |
| Faba bean  (*Vicia faba* L.) | **Growing:** Planting density 270 plants/m². 20/4 h day/night photoperiod; light intensity 500 μmol/m²/s at shelf level. 21/16°C day/night. Treatment with 10^-5^ M 6-BAP solution by spraying (10 ml/plant) between 9-10 AM, applied 4 days after flowering. Short-term temperature drop to 8/4°C day/night for 2 days starting 4 days after flowering | Flowering at 30-32 days | Time of one generation and generations per year — not provided | (Mobini et al., 2020) |
| Grass pea (*Lathyrus sativus* L.) | **Growing:** 22/2 h day/night photoperiod with 1 h sunrise/sunset simulation; light intensity 440-650 μmol/m²/s at adult plant level. 22/17°C day/night with gradual changes during sunrises/sunsets. Humidity 70% | Flowering at 31 days | 80 days; 4,5 generations | (Ghosh et al., 2018) |
| Lentils  (*Lens culinaris* Medik.) | **Growing:** 20/4 h day/night photoperiod  **Generation turnover:** harvest of immature seeds, dormancy breaking using 100 μmol GA | Flowering at 25-38 days | 56 days; 6 generations | (Lulsdorf and Banniza, 2018) |
|  | **Growing:** Planting density >300 seeds/m². 18/6 h day/night photoperiod; LED lighting; 410-730 nm spectrum; light intensity 74-93 μmol/m²/s (4000-5000 lux). 23-25/14-16°C day/night | Flowering at 27 days | 62-76 days;  6 generations | (Mitache et al., 2024a, 2024b) |
| Pea  (*Pisum sativum* L.) | **Growing:** Planting density 266-531 plants/m². Application of 0.6 μM flurprimidol at the three-leaf stage. 20/4 h day/night photoperiod, light intensity 500 μmol/m²/s. 21/16°C day/night  **Generation turnover:** embryo rescue at 14 days after flowering | Flowering at 33.4 days | 68.4 days;  5 generations | (Mobini and Warkentin, 2016) |
|  | **Growing:** 260 ml pots. 22/2 h day/night photoperiod with 1 h sunrise/sunset simulation. Light intensity 360-380 μmol/m²/s at shelf level. 22/17°C day/night, gradual changes during sunrises/sunsets. Humidity 70%  **Maturation:** irrigation stopped 6 weeks after sowing. Pea seeds scarified before sowing. Germinated for 5 days on moist filter paper in darkness | Flowering at 26.4±1.9 days | 51 days;  6 generations | (Watson et al., 2018) |
|  | **Growing:** Planting density 266 plants/m². 22/2 h day/night photoperiod; fluorescent lamps. 20±2°C. Application of 0.6 μM flurprimidol at 3-leaf stage  **Generation turnover:** seed harvest at 24 days after flowering | Flowering at 46-57 days | 70-81 days;  4.5-5.2 generations | (Cazzola et al., 2020) |
| Peanut  (*Arachis*  *hypogaea* L.) | **Growing:** 24 h light photoperiod; light intensity 450 μmol/m²/s. Day temperature 28±3°C, night temperature may drop to 17±3°C. Humidity 65% | Flowering at 25-27 days | 89 days; 4 generations | (O’Connor et al., 2013) |
| Pigeon pea  (*Cajanus cajan*  (L.) Huth.) | **Growing:** 13/11 h day/night photoperiod during vegetative phase, 8/16 h day/night photoperiod during flowering, 13/11 h day/night during maturation. LED lighting with far-red light during flowering. 32-35/22-25°C day/night during vegetative and maturation phases, 25-27/16-18°C day/night during flowering. Humidity 60-70% | Flowering at 21-100 days | 61-170 days;  2-4 generations | (Gangashetty et al., 2024) |
| Soybean  (*Glycine max* (L.) Merrill) | **Growing:** 14/10 h day/night photoperiod; light intensity 220 μmol/m²/s at source. 30/25°C day/night. CO_2_ levels maintained at 400-600 ppm  **Generation turnover:** pods collected when color changes from green to yellow, dried for 8 days at 27°C | Flowering at 25 days | 70 days; 5 generations | (Nagatoshi and Fujita, 2019) |
|  | **Growing:** 10/14 h day/night photoperiod; blue light predominates over red; lacking far-red; light intensity 1000 μmol/m²/s. 28°C. Humidity 80-100%  **Generation turnover:** irrigation stopped 5 days before harvest. Collected pods dried at 37°C for 24 h | Flowering at 23 days | 77 days; 5 generations | (Jähne et al., 2020) |
|  | **Growing:** 12/12 h day/night photoperiod, LED lighting (red and blue lights), light intensity 1018 μmol/m²/s. 29/27°C day/night | Time of flowering not provided | 63-81 days; up to 5 generations | (Harrison et al., 2021) |
|  | **Growing:** high-density sowing. 9/15 h day/night photoperiod; LED lighting; intensity 506 μmol/m²/s 30 cm above soil. Temperature 25±2°C. Humidity 50±10% | Flowering at 27-30 days | 73 days; 5 generations | (Lee et al., 2023) |
| Vigna  (*Vigna unguiculata* [L.] Walp.) | **Growing:** 10/14 h day/night photoperiod; LED lighting; light intensity 230-420 μmol/m²/s. 22-23/25-29°C day/night. Humidity 70%/80% day/night  **Generation turnover:** pods dried 11 days after flowering in a drying oven at 39°C for 2 days with silica gel | Flowering at 32-44 days | 45-57 days;  7-8 generations | (Edet and Ishii, 2022) |
| ***Malvaceae*** | | | | |
| Cotton (*Gossypium* sp.) | **Growing:** 10 L pots, 2 plants/pot. 12/12 h day/night photoperiod; full-spectrum LED lighting; light intensity 300-650 μmol/m²/s at canopy level. 32/26°C day/night. Humidity 45%  **Generation turnover:** embryo isolation at 25-30 days after pollination | Flowering at 45-46 days | 79,5 days;  5 generations | (Wang et al., 2025) |
| ***Poaceae*** | | | | |
| Barley (spring)  (*Hordeum vulgare* L.) | **Growing:** 18 ml cell trays. Foliar calcium nitrate fertilization (1 g/L). 22/2 h day/night photoperiod with 1 h sunrise/sunset simulation; full-spectrum lighting (emphasizes on blue and far-red); light intensity 440-650 μmol/m²/s at canopy level. 22/17°C day/night, gradual temperature changes during sunrises/sunsets. Humidity 70%  **Generation turnover:** spikes harvested 14-21 days post-flowering following gradual irrigation cessation. Seeds dried at 28-35°C for 3-5 days, with dormancy broken by 0.5 mg/L GA treatment at 4°C for 3 days | Flowering at 37-38 days | 55-60 days;  6 generations | (Watson et al., 2018) |
|  | **Growing:** 80 ml cell trays. Weekly micronutrient foliar applications and tri-weekly compound fertilizer irrigation. 22/2 h day/night photoperiod with dawn/dusk simulation; light intensity 274-505 μmol/m²/s. 21-23°C. Humidity 45-60%  **Maturation:** at soft dough stage, irrigation reduces followed by spike drying at 28°C for 5 days. Seed dormancy broken by 0.5 mg/L GA treatment at 4°C for 3 days or 37°C pre-treatment for 6 days. Alternatively, through embryo culture | Heading at 39 days | 77 days (seed drying) or 60 days (embryo culture); up to 6 generations | (Marenkova et al., 2024) |
| Barley (winter) (*Hordeum vulgare* L.) | **Vernalization:** germinated seeds on soil surface. 22/2 h day/night photoperiod; full-spectrum lighting (enhanced red and blue); light intensity 100-1300 μmol/m²/s. Temperature 10°C  **Growing:** 22/2 h day/night photoperiod. Temperature 22°C day/17°C night | 28 days of vernalization, flowering at 50 days | 2,4 months; 5 generations | (Cha et al., 2022) |
|  | **Vernalization:** Seedlings. 23/1 h day/night photoperiod; light intensity 500 μmol/m²/s. 10°C. Humidity 80/90% day/night  **Growing:** 100-cell trays. 23/1 h day/night photoperiod; light intensity 500 μmol/m²/s. 25/22°C day/night. Humidity 60/75% day/night  **Generation turnover:** embryo isolation at 12 days post-flowering. Petri dishes with embryos vernalized similarly to germinating seeds | 1-4 weeks vernalization, 49,2-72,3 days | time of one generation— not provided; up to 7 generations | (Zheng et al., 2023) |
| Bermuda grass (*Cynodon dactylon* [L.] pers и *Cynodon transvaalensis* Burtt-Davy) | **Growing:** 22/2 h day/night photoperiod; metal halide sodium lamps. 20°C | Flowering at 36-44 days | Time of one generation and generations per year — not provided | (Ji et al., 2022) |
| Finger millet (*Eleusine coracana* L.) | **Growing:** Planting density 105 plants/1.5 sq.ft., 0.17% Hoagland’s No.2 solution. 9/15 h day/night photoperiod; LED lighting. 29±2°C. Humidity 70%  **Generation turnover:** gradual irrigation cessation, harvesting at physiological maturity | Flowering at 52.6 days | 68,6 days;  4-5 generations | (Sajja et al., 2025) |
| Oats (spring)  (*Avena sativa* L.) | **Growing:** 22/2 h day/night photoperiod; light intensity 450-500 μmol/m²/s. 20/16°C day/night  **Generation turnover:** panicle cutting at 21 days after flowering. Drying in containers with silica gel for 5 days. Dormancy breaking at 4°C for 3 days | Flowering at 44.9-61.7 days | 72 days;  3,2-4,9 generations | (González‐Barrios et al., 2021) |
|  | **Growing:** Planting density 150 seeds/pot. Sand as substrate. 22/2 h day/night photoperiod; metal halide lamps, light intensity 900 μmol/m²/s at shelf level. 23/21°C day/night | Flowering at 29.9±2.9 days | Time of one generation and generations per year — not provided | (Kigoni et al., 2023) |
| Oats (winter) (*Avena sativa* L.) | **Vernalization:** Seedlings. 23/1 h day/night photoperiod; light intensity 500 μmol/m²/s. 10°C. Humidity 80/90% day/night  **Growing:** 100-cell trays. 23/1 h day/night photoperiod; light intensity 500 μmol/m²/s. 25/22°C day/night. Humidity 60/75% day/night  **Generation turnover:** embryo isolation at 12 days post-flowering. Petri dishes with embryos vernalized similarly to germinating seeds | 4-5 weeks vernalization, 67,5-76,8 days | Time of one generation not provided; 5 generations | (Zheng et al., 2023) |
| Oats (sandy oats)  (*Avena strigosa* Schreb.) | **Growing:** 18 ml cell trays. foliar feeding with calcium nitrate (1 g/l). 22/2 h day/night photoperiod with 1 h sunrise and sunset simulations; full-spectrum lighting (emphasis on blue and far-red); light intensity at adult plant level 440-650 μmol/m²/s. 22/17°C day/night, gradual increase/decrease during sunrise/sunset. Humidity 70% | Flowering at 52 days | 100 days; up to 3 generations | (Ghosh et al., 2018) |
| Rice  (*Oryza sativa* L.) | **Growing:** 10/14 h day/night photoperiod; light intensity 574 μmol/m²/s; presence of far-red light | Flowering at 60 days | Time of one generation and generations per year — not provided | (Jähne et al., 2020) |
|  | **Growing:** seeds are pre-incubated at 40-45°C for 48 h before sowing. 250 ml pots with two plants per pot. Continuous lighting for the first 15 days, followed by 10/14 h day/night. Light intensity 800 μmol/m²/s. Spectral composition: red and blue (2:1 ratio), green, yellow, and far-red lights. 32/30°C day/night. Humidity 65%  **Generation turnover:** harvesting immature seeds 13-15 days after flowering, drying at 38°C. Dormancy breaking by 80 ppm GA treatment at 16-18°C for 12 h | Flowering at 52-60 days | 67-75 days; 4-5 generations | (Kabade et al., 2024) |
|  | **Growing**: Planting density 700 plants/m². Substrate composed of manure and soil (1:1 ratio). 13/11 h day/night photoperiod during early growth stages, then 8/16 h day/night. Light intensity 750-800 μmol/m²/s at plant height. 30-32/23-25°C day/night. Root fertilization with NPK and Zn solution  **Generation turnover:** spike cutting at 15 days after flowering, drying at 38°C for 24 h. Treatment of dried seeds with 100 ppm GA or 2% CaCl_2_ solution for 20 h at 16-18°C, followed by sowing | Flowering occurs at 55 days | 68-75 days; 4-5 generations | (Sandhu et al., 2024) |
| Stiff brome (*Brachypodium distachyon* L.) | **Growing:** 18 ml cell trays. Foliar feeding with calcium nitrate (1 g/l). 22/2 h day/night photoperiod with 1-hour sunrise/sunset simulations; full-spectrum lighting (emphasis on blue and far-red); light intensity 440-650 μmol/m²/s. 22/17°C day/night, gradual changes during sunrise/sunset. Humidity 70%  **Maturation:** spike cutting at 14-21 days post-flowering, gradual irrigation cessation over 1 week, seed drying at 28-35°C for 3-5 days. Dormancy breaking by 0.5 mg/L GA treatment at 4°C for 3 days | Heading at 26 days | 50 days;  7 generations | (Watson et al., 2018) |
| Triticale (spring) (×*Triticosecale* Wittm.) | **Growing:** 72-cell trays. 22/2 h day/night photoperiod. 22/17°C day/night  **Generation turnover:** immature seeds drying at 35°C for 4 days. Dormancy breaking at 4°C for 3 days | Flowering at 38 days | 60 days; up to 6 generations | (Cha et al., 2021) |
|  | **Growing:** 110 ml cell trays. 22/2 h day/night photoperiod; light intensity 330 μmol/m²/s; R/FR = 0.3. 25-26°C. Humidity 35-45%.  **Generation turnover:** spike cutting at 20 days post-flowering, followed by spike drying at 28°C for 7-10 days. Seed dormancy broken by 0.5 mg/L GA treatment at 4°C for 3 days. Alternatively, through embryo culture at 15 days post-flowering | Flowering at 33.9 days | 64 days (seed drying) or 49 days (embryo culture); 6 generations | (Blinkov et al., 2025) |
| Triticale (winter) (×*Triticosecale* Wittm.) | **Vernalization:** Seedlings. 23/1 h day/night photoperiod; light intensity 500 μmol/m²/s. 10°C. Humidity 80/90% day/night  **Growing:** 100-cell trays. 23/1 h day/night photoperiod; light intensity 500 μmol/m²/s. 25/22°C day/night. Humidity 60/75% day/night  **Generation turnover:** embryo isolation at 12 days post-flowering. Petri dishes with embryos vernalized similarly to germinating seeds | 5 weeks vernalization, flowering at 73,3-77,1 days | Time of one generation— not provided; 5 generations | (Zheng et al., 2023) |
| Wheat (spring common wheat (*Triticum aestivum* L.) and spring durum wheat (*Triticum durum* Desf.) | **Growing:** 18 ml cell trays. 22/2 h day/night photoperiod with 1 h dawn/dusk simulation; full-spectrum lighting (emphasis on blue and far-red); light intensity at plant canopy level 440-650 μmol/m²/s. 22/17°C day/night, gradual transitions during dawn/dusk periods. Humidity 70%. Foliar calcium nitrate fertilization (1 g/L)  **Generation turnover:** spike collection 14-21 days post-flowering, gradual irrigation cessation over one week prior to harvest. Seed drying at 28-35°C for 3-5 days. 0.5 mg/L GA solution at 4°C for 3 days for seed dormancy breaking | Flowering at 35.7–63 days | 56-80 days;  5-6 generations | (Ghosh et al., 2018; Watson et al., 2018) |
| Wheat (winter common wheat) (*Triticum aestivum* L.)) | **Vernalization:** germinating seeds. 22/2 h day/night photoperiod. 4.5/9°C day/night  **Growing:** 22/2 h day/night photoperiod; LED full-spectrum lighting (enhanced red and blue), light intensity at shelf level 300 μmol/m²/s. 22°C. Humidity 70%. Tillers removed | 14-25 days vernalization, flowering at 58 days | 83 days;  up to 5 generations | (Song et al., 2021) |
|  | **Vernalization:** germinated seeds on soil surface. 22/2 h day/night photoperiod; full-spectrum lighting (enhanced red and blue); light intensity 100-1300 μmol/m²/s. 10°C  **Growing:** 22/2 h day/night photoperiod. 22°C day/17°C night | 28 days vernalization, flowering at 46.8-80 days | 82.4 days;  up to 5 generations | (Cha et al., 2022) |
|  | **Vernalization:** 3-leaf stage. 12/12 h day/night photoperiod; light intensity 100 μmol/m²/s. 4°C. Humidity 60-65%  **Growing:** 22/2 h day/night photoperiod; full-spectrum lighting (R:FR = 8); DLI 28.51-28.80. 22°C. Humidity 60-65% | 9 weeks vernalization, flowering at 110-120 days | 135-145 days; up to 3 generations | (Ficht et al., 2023) |
|  | **Vernalization:** 1-leaf stage. 12/12 h day/night photoperiod; light intensity 150-280 μmol/m²/s. 4°C  **Growing:** 22/2 h day/night photoperiod; light intensity 315-350 μmol/m²/s. 25/22°C day/night  **Generation turnover:** spike collection at 10 days post-flowering. Drying at 50°C for 3 days | 6 weeks vernalization, flowering at 83.6±4.3 days | 87 days;  4 generations | (Schoen et al., 2023) |
|  | **Vernalization:** Seedlings. 23/1 h day/night photoperiod; light intensity 500 μmol/m²/s. 10°C. Humidity 80/90% day/night  **Growing:** 100-cell trays. 23/1 h day/night photoperiod; light intensity 500 μmol/m²/s. 25/22°C day/night. Humidity 60/75% day/night  **Generation turnover:** embryo isolation at 12 days post-flowering. Petri dishes with embryos vernalized similarly to germinating seeds | 2-5 weeks vernalization, time of flowering— not provided | 55,6-84,6 days; up to 6 generations | (Zheng et al., 2023) |
| ***Solanaceae*** | | | | |
| Pepper  (*Capsicum annuum*  L.) | **Seedling growing:** Trays. 16/8 h day/night photoperiod. 25/20°C day/night  **Adult plant growing:** Plants are transplanted into pots. 20/4 h day/night photoperiod; fluorescent lamps; R:FR = 0.3. 27/24°C day/night | Flowering at 36 days | 110 days; 3 generations | (Choi et al., 2023) |
| Hot pepper  (*Capsicum* spp.) | **Growing:** 12/12 h day/night photoperiod; LED lighting; R:FR = 2.1; light intensity 420 μmol/m²/s | Flowering at 40 days | 82 days;  more than 4 generations | (Liu et al., 2022) |
| Tomato  (*Solanum lycopersicum* L.) | **Growing:** 6 L pots. 16/8 h day/night photoperiod; fluorescent lamps. 25/18°C day/night. Сold stress treatment at 14°C for 8 days during cotyledon stage. 10 g potassium sulfate per pot at planting and before fruit maturation  **Generation turnover:** embryo culture | Flowering at 51-53 days | Embryo isolation at 84-86 days; 4 generations | (Gimeno-Páez et al., 2025) |
|  | Induced overexpression of *FLOWERING LOCUS T* (*FLT*) from Arabidopsis through infiltration of tomato cotyledons at 14 days after germination with *Agrobacterium tumefaciens* strain GV3101 night culture suspension carrying viral vector pGR107 expressing *FLT* | Flowering and maturation 14-21 days earlier compared to uninfected plants | | (Deng et al., 2024) |

# Reference

Blinkov, A. O., Nagamova, V. M., Minkova, Y. V., Svistunova, N. Yu., Radzeniece, S., Kocheshkova, A. A., et al. (2025). The reduction of triticale generation time under speed breeding conditions by increasing the amounts of far-red light in the optical radiation. *Vavilov Journal of Genetics and Breeding*, In Press.

Cazzola, F., Bermejo, C. J., Guindon, M. F., and Cointry, E. (2020). Speed breeding in pea (*Pisum sativum* L.), an efficient and simple system to accelerate breeding programs. *Euphytica* 216, 178. doi: 10.1007/s10681-020-02715-6

Cha, J.-K., O’Connor, K., Alahmad, S., Lee, J.-H., Dinglasan, E., Park, H., et al. (2022). Speed vernalization to accelerate generation advance in winter cereal crops. *Molecular Plant* 15, 1300–1309.

Cha, J.-K., Park, M.-R., Shin, D., Kwon, Y., Lee, S.-M., Ko, J.-M., et al. (2021). Growth Characteristics of Triticale under Long-Day Photoperiod for Rapid Generation Advancement. *Korean J. Breed. Sci.* 53, 200–205. doi: 10.9787/KJBS.2021.53.3.200

Choi, H., Back, S., Kim, G. W., Lee, K., Venkatesh, J., Lee, H. B., et al. (2023). Development of a speed breeding protocol with flowering gene investigation in pepper (*Capsicum annuum*). *Front. Plant Sci.* 14, 1151765. doi: 10.3389/fpls.2023.1151765

Çil, A. N. (2023). Breeding Sunflower (Helianthus annuus) Assisted With Speed Breeding & Drough Tolerance Tests. *Selcuk Journal of Agriculture and Food Sciences* 37, 474–486.

D’Angelo, C. J., and Goldman, I. L. (2019). Annualization of the long day onion breeding cycle through threshold vernalization and dormancy disruption. *Crop Breeding, Genetics and Genomics* 1.

Deng, Y., Yarur-Thys, A., and Baulcombe, D. C. (2024). Virus-induced overexpression of heterologous *FLOWERING LOCUS T* for efficient speed breeding in tomato. *Journal of Experimental Botany* 75, 36–44. doi: 10.1093/jxb/erad369

Edet, O. U., and Ishii, T. (2022). Cowpea speed breeding using regulated growth chamber conditions and seeds of oven-dried immature pods potentially accommodates eight generations per year. *Plant Methods* 18, 106.

Ficht, A., Bruch, A., Rajcan, I., Pozniak, C., and Lyons, E. M. (2023). Evaluation of the impact of photoperiod and light intensity on decreasing days to maturity in winter wheat. *Crop Science* 63, 812–821.

Gangashetty, P. I., Belliappa, S. H., Bomma, N., Kanuganahalli, V., Sajja, S. B., Choudhary, S., et al. (2024). Optimizing speed breeding and seed/pod chip based genotyping techniques in pigeonpea: A way forward for high throughput line development. *Plant Methods* 20, 27. doi: 10.1186/s13007-024-01155-w

Gaoua, O., Arslan, M., and Obedgiu, S. (2025). Speed breeding advancements in safflower (*Carthamus tinctorius* L.): a simplified and efficient approach for accelerating breeding programs. *Mol Breeding* 45, 13. doi: 10.1007/s11032-024-01530-4

Ghosh, S., Watson, A., Gonzalez-Navarro, O. E., Ramirez-Gonzalez, R. H., Yanes, L., Mendoza-Suárez, M., et al. (2018). Speed breeding in growth chambers and glasshouses for crop breeding and model plant research. *Nat Protoc* 13, 2944–2963. doi: 10.1038/s41596-018-0072-z

Gimeno-Páez, E., Prohens, J., Moreno-Cerveró, M., De Luis-Margarit, A., Díez, M. J., and Gramazio, P. (2025). Agronomic treatments combined with embryo rescue for rapid generation advancement in tomato speed breeding. *Horticultural Plant Journal* 11, 239–250. doi: 10.1016/j.hpj.2023.06.006

González‐Barrios, P., Bhatta, M., Halley, M., Sandro, P., and Gutiérrez, L. (2021). Speed breeding and early panicle harvest accelerates oat (*Avena sativa* L.) breeding cycles. *Crop Science* 61, 320–330. doi: 10.1002/csc2.20269

Harrison, D., Da Silva, M., Wu, C., De Oliveira, M., Ravelombola, F., Florez‐Palacios, L., et al. (2021). Effect of light wavelength on soybean growth and development in a context of speed breeding. *Crop Science* 61, 917–928. doi: 10.1002/csc2.20327

Jähne, F., Hahn, V., Würschum, T., and Leiser, W. L. (2020). Speed breeding short-day crops by LED-controlled light schemes. *Theoretical and Applied Genetics* 133, 2335–2342.

Ji, M., Wang, G., Liu, X., Li, X., Xue, Y., Amombo, E., et al. (2022). The extended day length promotes earlier flowering of bermudagrass. *PeerJ* 10, e14326. doi: 10.7717/peerj.14326

Kabade, P. G., Dixit, S., Singh, U. M., Alam, S., Bhosale, S., Kumar, S., et al. (2024). SpeedFlower: a comprehensive speed breeding protocol for indica and japonica rice. *Plant Biotechnology Journal* 22, 1051–1066.

Kigoni, M., Choi, M., and Arbelaez, J. D. (2023). ‘Single-Seed-SpeedBulks:’a protocol that combines ‘speed breeding’with a cost-efficient modified single-seed descent method for rapid-generation-advancement in oat (*Avena sativa* L.). *Plant Methods* 19, 92.

Kuroda, Y., Kuranouchi, T., Okazaki, K., Takahashi, H., and Taguchi, K. (2024). Biennial sugar beets capable of flowering without vernalization treatment. *Genet Resour Crop Evol* 71, 823–834. doi: 10.1007/s10722-023-01662-0

Lee, D., Han, K., Kim, J. H., Jun, T.-H., and Lee, J. S. (2023). Development of Speed-Breeding System for Korean Soybean Varieties [*Glycine max* a (L.) Merr] Using LED Light Source. *Plant Breed. Biotech.* 11, 49–55. doi: 10.9787/PBB.2023.11.1.49

Liu, K., He, R., He, X., Tan, J., Chen, Y., Li, Y., et al. (2022). Speed Breeding Scheme of Hot Pepper through Light Environment Modification. *Sustainability* 14, 12225. doi: 10.3390/su141912225

Lulsdorf, M. M., and Banniza, S. (2018). Rapid generation cycling of an F_2_ population derived from a cross between *Lens culinaris* Medik. and *Lens ervoides* (Brign.) Grande after aphanomyces root rot selection. *Plant Breeding* 137, 486–491. doi: 10.1111/pbr.12612

Marenkova, A. G., Blinkov, A. O., Radzeniece, S., Kocheshkova, A. A., Karlov, G. I., Lavygina, V. A., et al. (2024). Testing and Modification of the Protocol for Accelerated Growth of Malting Barley under Speed Breeding Conditions. *Nanobiotechnology Reports* 19, 808–814.

Mitache, M., Baidani, A., Bencharki, B., and Idrissi, O. (2024a). Exploring the impact of light intensity under speed breeding conditions on the development and growth of lentil and chickpea. *Plant Methods* 20, 30. doi: 10.1186/s13007-024-01156-9

Mitache, M., Baidani, A., Zeroual, A., Bencharki, B., and Idrissi, O. (2024b). Rapid generation advancement through speed breeding in lentil (*Lens culinaris* Medik.). *Crop Breeding and Applied Biotechnology* 24, e48632435.

Mobini, S. H., and Warkentin, T. D. (2016). A simple and efficient method of in vivo rapid generation technology in pea (*Pisum sativum* L.). *In Vitro Cellular & Developmental Biology-Plant* 52, 530–536.

Mobini, S., Khazaei, H., Warkentin, T. D., and Vandenberg, A. (2020). Shortening the generation cycle in faba bean (*Vicia faba*) by application of cytokinin and cold stress to assist speed breeding. *Plant Breeding* 139, 1181–1189.

Nagatoshi, Y., and Fujita, Y. (2019). Accelerating Soybean Breeding in a CO_2_-Supplemented Growth Chamber. *Plant and Cell Physiology* 60, 77–84. doi: 10.1093/pcp/pcy189

O’Connor, D. J., Wright, G. C., Dieters, M. J., George, D. L., Hunter, M. N., Tatnell, J. R., et al. (2013). Development and Application of Speed Breeding Technologies in a Commercial Peanut Breeding Program. *Peanut Science* 40, 107–114. doi: 10.3146/PS12-12.1

Sajja, S., Pranati, J., Shyamala, S., Vinutha, K. S., Reddy, R., Joshi, P., et al. (2025). Rapid Ragi: A speed breeding protocol for finger millet. *Plant Methods* 21, 84.

Samineni, S., Sen, M., Sajja, S. B., and Gaur, P. M. (2020). Rapid generation advance (RGA) in chickpea to produce up to seven generations per year and enable speed breeding. *The Crop Journal* 8, 164–169. doi: 10.1016/j.cj.2019.08.003

Sandhu, N., Singh, J., Pruthi, G., Verma, V. K., Raigar, O. P., Bains, N. S., et al. (2024). SpeedyPaddy: a revolutionized cost-effective protocol for large scale offseason advancement of rice germplasm. *Plant Methods* 20, 109. doi: 10.1186/s13007-024-01235-x

Schilling, S., Melzer, R., Dowling, C. A., Shi, J., Muldoon, S., and McCabe, P. F. (2023). A protocol for rapid generation cycling (speed breeding) of hemp (*Cannabis sativa*) for research and agriculture. *The Plant Journal* 113, 437–445.

Schoen, A., Wallace, S., Holbert, M. F., Brown‐Guidera, G., Harrison, S., Murphy, P., et al. (2023). Reducing the generation time in winter wheat cultivars using speed breeding. *Crop Science* 63, 2079–2090.

Somody, G., and Molnár, Z. (2025). Flowering Synchronization Using Artificial Light Control for Crossbreeding Hemp (*Cannabis sativa* L.) with Varied Flowering Times. *Plants* 14, 594. doi: 10.3390/plants14040594

Somody, G., Molnár, Z., and Lakatos, E. (2024). Possibilities of rapid generation cycling of hemp (*Cannabis sativa* L.) for the stabilization of recessive traits. *BIO Web Conf.* 125, 01012. doi: 10.1051/bioconf/202412501012

Song, Y., Duan, X., Wang, P., Li, X., Yuan, X., Wang, Z., et al. (2021). Comprehensive speed breeding: a high‐throughput and rapid generation system for long‐day crops. *Plant Biotechnology Journal* 20, 13.

Stetter, M. G., Zeitler, L., Steinhaus, A., Kroener, K., Biljecki, M., and Schmid, K. J. (2016). Crossing Methods and Cultivation Conditions for Rapid Production of Segregating Populations in Three Grain Amaranth Species. *Front. Plant Sci.* 7. doi: 10.3389/fpls.2016.00816

Taku, M., Saini, M., Kumar, R., Debbarma, P., Rathod, N. K. K., Onteddu, R., et al. (2024). Modified speed breeding approach reduced breeding cycle to less than half in vegetable soybean [*Glycine max* (L.) Merr.]. *Physiol Mol Biol Plants* 30, 1463–1473. doi: 10.1007/s12298-024-01503-z

Taku, M., Saini, M., Kumar, R., Rathod, N. K. K., Reshma, O., Yadav, M., et al. (2025). Rapid Development of Lipoxygenase‐2 Free Vegetable Soybean Genotypes (*Glycine max* (L.) Merill) Through Molecular Breeding Under Controlled Environment. *Plant Breeding*, pbr.13267. doi: 10.1111/pbr.13267

Wang, G., Sun, Z., Yang, J., Ma, Q., Wang, X., Ke, H., et al. (2025). The speed breeding technology of five generations per year in cotton. *Theoretical and Applied Genetics* 138, 79.

Watson, A., Ghosh, S., Williams, M. J., Cuddy, W. S., Simmonds, J., Rey, M.-D., et al. (2018). Speed breeding is a powerful tool to accelerate crop research and breeding. *Nature plants* 4, 23–29.

Zheng, Z., Gao, S., Wang, H., and Liu, C. (2023). Shortening generation times for winter cereals by vernalizing seedlings from young embryos at 10 degree Celsius. *Plant Breeding* 142, 202–210. doi: 10.1111/pbr.13074
